# Radiomics of computed tomography and magnetic resonance imaging in renal cell carcinoma—a systematic review and meta-analysis

**DOI:** 10.1007/s00330-020-06666-3

**Published:** 2020-02-14

**Authors:** Stephan Ursprung, Lucian Beer, Annemarie Bruining, Ramona Woitek, Grant D Stewart, Ferdia A Gallagher, Evis Sala

**Affiliations:** 1 Department of Radiology, School of Clinical Medicine, University of Cambridge, Cambridge, UK; 2 Cancer Research UK Cambridge Centre, University of Cambridge, Cambridge, UK; 3 Department of Biomedical Imaging and Image-guided Therapy, Medical University of Vienna, Vienna, Austria; 4 Department of Radiology, Netherlands Cancer Institute, Amsterdam, The Netherlands; 5 Department of Surgery, School of Clinical Medicine, University of Cambridge, Cambridge, UK

**Keywords:** Carcinoma, renal cell, Angiomyolipoma, Machine learning, Quality improvement, Systematic review

## Abstract

**Objectives:**

(1) To assess the methodological quality of radiomics studies investigating histological subtypes, therapy response, and survival in patients with renal cell carcinoma (RCC) and (2) to determine the risk of bias in these radiomics studies.

**Methods:**

In this systematic review, literature published since 2000 on radiomics in RCC was included and assessed for methodological quality using the Radiomics Quality Score. The risk of bias was assessed using the Quality Assessment of Diagnostic Accuracy Studies tool and a meta-analysis of radiomics studies focusing on differentiating between angiomyolipoma without visible fat and RCC was performed.

**Results:**

Fifty-seven studies investigating the use of radiomics in renal cancer were identified, including 4590 patients in total. The average Radiomics Quality Score was 3.41 (9.4% of total) with good inter-rater agreement (ICC 0.96, 95% CI 0.93–0.98). Three studies validated results with an independent dataset, one used a publically available validation dataset. None of the studies shared the code, images, or regions of interest. The meta-analysis showed moderate heterogeneity among the included studies and an odds ratio of 6.24 (95% CI 4.27–9.12; *p* < 0.001) for the differentiation of angiomyolipoma without visible fat from RCC.

**Conclusions:**

Radiomics algorithms show promise for answering clinical questions where subjective interpretation is challenging or not established. However, the generalizability of findings to prospective cohorts needs to be demonstrated in future trials for progression towards clinical translation. Improved sharing of methods including code and images could facilitate independent validation of radiomics signatures.

**Key Points:**

• *Studies achieved an average Radiomics Quality Score of 10.8%. Common reasons for low Radiomics Quality Scores were unvalidated results, retrospective study design, absence of open science, and insufficient control for multiple comparisons.*

• *A previous training phase allowed reaching almost perfect inter-rater agreement in the application of the Radiomics Quality Score.*

• *Meta-analysis of radiomics studies distinguishing angiomyolipoma without visible fat from renal cell carcinoma show moderate diagnostic odds ratios of 6.24 and moderate methodological diversity.*

**Electronic supplementary material:**

The online version of this article (10.1007/s00330-020-06666-3) contains supplementary material, which is available to authorized users.

## Introduction

Radiological practice relies largely on the subjective interpretation of imaging data by an expert radiologist. Reports will therefore be dependent on reader experience. Quantitative, reader independent imaging markers may supplement expert opinion and increase diagnostic, predictive, and prognostic accuracy [1]. Radiomics includes a number of strategies aimed at converting medical images to quantitative, minable, high-dimensional data. These include histogram, texture, and shape analysis that extract information from imaging data which may not be visible to the human eye [2, 3]. In recent years, increased interest in the use of radiomics in oncological imaging has led to its application as a tool to derive diagnostic, predictive, and prognostic information from routine clinical imaging [4]. Despite extensive use in research and reports linking CT and MR texture to lesion characterization, survival, and perioperative outcome in a number of malignancies, translation into clinical practice has not yet occurred [5].

**Fig. 1:**
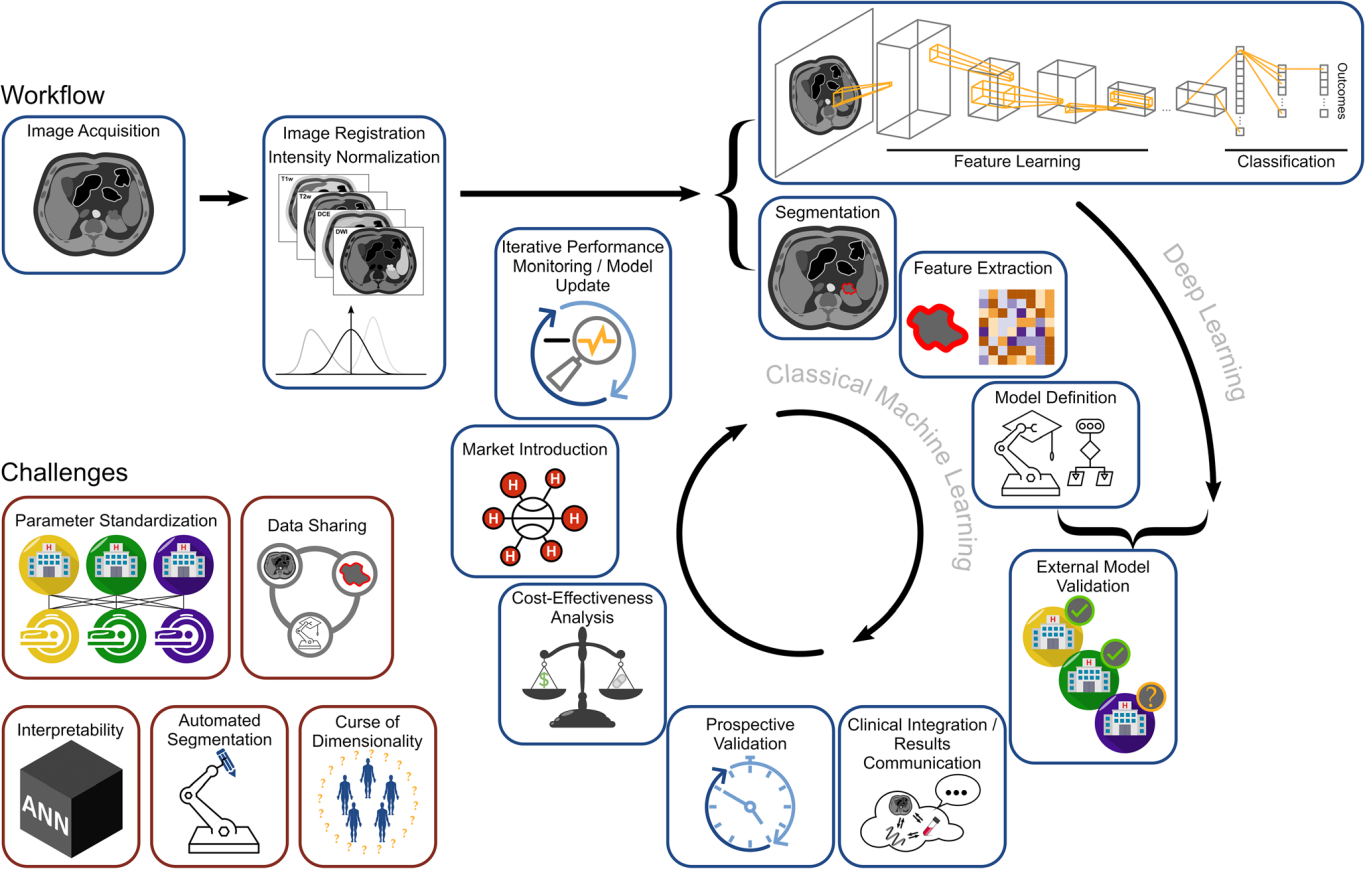
Pathway for the development of radiomics algorithms and challenges in clinical translation. In addition to image acquisition and image registration, non-quantitative MRI sequences may undergo intensity normalization to reduce intra- and inter-patient heterogeneity. Subsequently, either classical machine learning algorithms or deep learning are employed to define diagnostic, prognostic, or predictive models. These models require external validation, ensuring transferability of results between sites and MR scanners before prospective validation and demonstration of cost-effectiveness can enable these diagnostic support systems to enter clinical practice. Continuous monitoring is required to detect deteriorating model performance to trigger re-training and model update as image acquisition evolves. ANN: artificial neural network

Renal cell carcinoma (RCC) is newly diagnosed in 338,000 patients annually worldwide and incidence varies widely with the highest incidence in Northern America, Europe, Australia, and New Zealand [6]. Most countries have seen a rise in incidence over the past decades, which has been attributed to the increasing use of cross-sectional imaging and subsequent incidental diagnosis [7]. Increasing diagnosis of small renal masses carries the risk of overtreatment resulting in benign histology in 10–30% of all resected tumors [8, 9]. While CT is the mainstay of diagnostic imaging in RCC, MRI has become a valuable problem-solving tool. Owing to its improved soft-tissue-contrast, MRI outperforms CT in the evaluation of indeterminate cystic masses (Bosniak 2F and 3, malignancy in 10% and 50% respectively) [10], local invasion, and intra-vascular extension [11]. Still, the differentiation of benign renal lesions, especially oncocytoma and angiomyolipoma without visible fat (AMLwvf), from RCC can be challenging by subjective radiological image interpretation [12]. Quantitative image analysis may reveal radiomic signatures diagnostic of renal tumor subtype and aggressiveness or predictive of response to targeted treatment, therefore, aiding treatment stratification. However, for imaging markers including texture-based metrics to cross the translational gap between an exploratory research tool and a clinically applicable diagnostic algorithm, technical validity, biological validity, qualification, and cost-effectiveness need to be established (Fig. 1) [13].

This systematic review aims to establish whether the methodological quality of prospective and retrospective studies published on radiomics in cross-sectional imaging of renal tumors for diagnostic, predictive, and prognostic purposes poses barriers to effective clinical translation. A meta-analysis of the use of texture-based models for the discrimination of AMLwvf and RCC shall assess the ability of proposed models to answer this clinically relevant question.

## Methods

This systematic review was conducted according to the PRISMA-DTA (Preferred Reporting Items for Systematic Reviews and Meta-analysis for Diagnostic Test Accuracy) statement [14]. The review protocol is available through PROSPERO (CRD 42018115263). The electronic databases PubMed, EMBASE, and Web of Science were searched for primary publications in English assessing texture analysis in RCC in CT or MRI published after 01/01/2000. The databases were last searched on the 30/10/2018. The search term consisted of (textural OR radiomics OR texture OR histogram) AND (kidney OR renal) AND (“computed tomography” OR CT or “magnetic resonance” OR MRI OR MR).

A single researcher with 2 years of post-graduate experience in medical image analysis (S.U.) screened titles and abstracts to determine eligibility. Articles in which texture analysis was employed for diagnostic, predictive, or prognostic purposes on CT or MR images of RCC were obtained in full for further evaluation. Contact with the authors was sought if the full-text version was not accessible otherwise. Studies were excluded if they were case reports, conference abstracts, or short communications because they do not provide sufficient information to assess the methodological quality. The reference lists of included studies were screened for additional, potentially eligible articles. Uncertainties were resolved in consensus between SU, LB, and AB.

The Radiomics Quality Score (RQS) and the Quality Assessment of Diagnostic Accuracy Studies (QUADAS-2) were used to assess the methodological quality of the included studies and the risk of bias on the study level, respectively [15, 16]. The RQS is a recently proposed tool to measure the methodological rigor of radiomics studies. It interrogates image acquisition, radiomics features extraction, data modeling, model validation, and data sharing. Each of the 16 dimensions (Table 1) of the score is rated resulting in a sum of points ranging from − 8 to 36 with − 8 to 0 defined as 0% and 36 defined as 100% [15]. The QUADAS-2 tool assesses the presence of bias in the domains of “patient selection,” “index test,” “reference standard,” and “flow and timing.” The tool can be tailored to the specific research question through signaling questions for risks of bias which are specific to the individual research question [16].

**Table 1:** Elements of the RQS as described by Lambin et al [15] and average rating achieved by the studies included in this systematic review

RQS scoring item	Interpretation	Average
Image Protocol	+ 1 for well documented protocols, + 1 for publicly available protocols	0.48
Multiple Segmentations	+ 1 if segmented multiple times (different physicians, algorithms, or perturbation of regions of interest)	0.38
Phantom Study	+ 1 if texture phantoms were used for feature robustness assessment	0.00
Multiple Time Points	+ 1 multiple time points for feature robustness assessment	0.01
Feature Reduction	− 3 if nothing, + 3 if either feature reduction or correction for multiple testing	0.23
Non Radiomics	+ 1 if multivariable analysis with non-radiomics features	0.15
Biological Correlates	+ 1 if present	0.98
Cut-off	+ 1 if cutoff either pre-defined or at median or continuous risk variable reported	0.11
Discrimination and Resampling	+ 1 for discrimination statistic and statistical significance, + 1 if resampling applied	0.92
Calibration	+ 1 for calibration statistic and statistical significance, +1 if resampling applied	0.04
Prospective	+ 7 for prospective validation within a registered study	0.98
Validation	− 5 if no validation/+ 2 for internal validation/+ 3 for external validation/+ 4 two external validation datasets or validation of previously published signature/+ 5 validation on ≥ 3 datasets from > 1 institute	−4.61
Gold Standard	+ 2 for comparison to gold standard	1.73
Clinical Utility	+ 2 for reporting potential clinical utility	1.91
Cost-effectiveness	+ 1 for cost-effectiveness analysis	0.00
Open Science	+ 1 for open-source scans, + 1 for open-source segmentations, + 1 for open-source code, + 1 open-source representative segmentations and features	0.02

*RQS*: Radiomics Quality Score

During a training phase, the three reviewers (doctoral student with 2 years of post-graduate experience in medical image analysis (S.U.), a radiologist in the 4th year of training (L.B.), and a board-certified radiologist with 8 years of experience (A.B.)) independently extracted study data from two randomly chosen articles into a structured data collection instrument generated based on RQS and QUADAS-2. Disagreements were discussed in order to achieve a shared understanding of each parameter. Subsequently, at least two raters assessed and rated each study independently and recorded these on the data collection instrument. The data collection instrument can be found in supplementary Table S1.

Statistical analysis was conducted using R language for statistical computing [17]. Analyses were performed using the metafor, irr, and raters packages [18]. Unless otherwise specified, the average rating of all raters is reported. Inter-rater agreement for single items of the RQS was calculated using a modified Fleiss kappa statistic for ordinal variables [19]. A 95% confidence interval was derived from a Monte Carlo test and bootstrap procedure over 1000 iterations. *P* values for the null hypothesis that agreement resulted from chance alone were calculated. The interclass correlation coefficient (ICC) was determined to describe inter-rater agreement for the summed RQS using a single source, two-way random effects model determining absolute agreement between raters.

As pre-defined in the review protocol, studies would be included in a meta-analysis of a large enough subset of the included studies if a similar clinical question was assessed repeatedly. Upon review of the study population, the differentiation of lesions defined as either fat poor AML, AMLwvf, or AML without macroscopic fat from malignant renal tumors was addressed repeatedly. These studies were included in the meta-analysis. Two-by-two contingency tables were extracted or reconstructed and odds ratios were calculated as effect size. A random effects model was used to calculate the summary effect size. If multiple texture models were reported in a study, only the one with the highest area under the receiver operating curve or the highest Youden’s J statistic, if no AUC was reported, was included. If data augmentation, the generation of new data through random transformation of existing cases, was performed, the augmented cases were not included in the meta-analysis. A funnel plot was constructed to visually assess the risk of publication bias and the trim and fill method was used to estimate the number of missing studies. *Q* and *I*^2^ were calculated to estimate the heterogeneity among the studies included in the meta-analysis. A more detailed description of the statistical methods can be found in the supplementary materials.

## Results

**Fig. 2:**
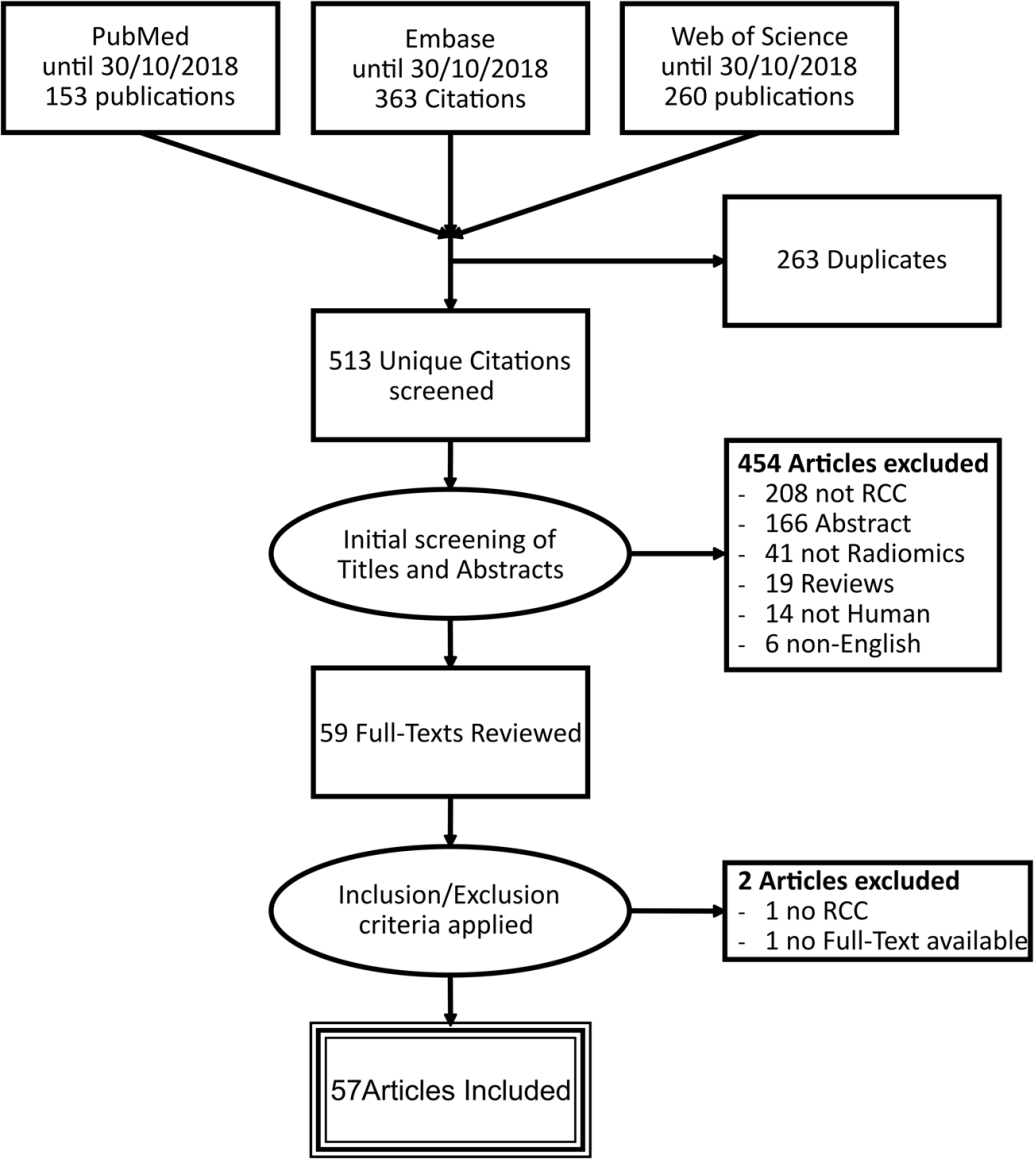
Study selection flowchart

The initial search yielded 776 articles of which 263 were duplicates. Of the remaining 513, 454 were rejected based on title and abstract. Of the 59 full-text manuscripts retrieved, 57 were included in the systematic review (Fig. 2). The articles employed radiomics-based diagnostic models to assess similar clinical questions repeatedly. The differentiation of benign and malignant lesions was investigated by 39% (22/57) of the articles while 27% (15/57) explored subtype differentiation and 21% (12/57) interrogated treatment response/outcome prediction. Tables S2 and S3 summarize study aims and characteristics, respectively.

The 57 studies reached a mean ± standard deviation RQS of 3.41 ± 4.43, median 4.5, interquartile range 6.17, and range − 4.0–16.6. The average percentage RQS was 9.4% with a maximum of 46%. The average rating for each dimension is summarized in Table 1, and the RQS for individual studies and individual ratings for each study are presented in Tables S2 and S4 respectively. Most studies applied discrimination statistics, included biological correlates, and addressed their potential clinical utility. Conversely, none of the studies included in this systematic review employed phantoms or assessed the cost-effectiveness of radiomics-based decision support systems. No study shared either segmentations or code publicly and only few assessed the repeatability of radiomics analysis at multiple time points, employed calibration statistics or a validation cohort. Only 39% (22/57) of the studies segmented the entire 3D tumor volume for texture analysis, and 91% (52/57) used manual segmentation. Inter-reader agreement was assessed in 32% (18/57) of the studies and found to be moderate to excellent for single features or radiomics signatures. Only a single study investigated the repeatability of radiomics measurements and found poor to good repeatability of histogram parameters of the transfer constant of dynamic contrast-enhanced MRI [20].

Studies included in this review extracted between four and 18,720 features (median 24) from two to 249 patients (median 61). The ratio between features and patients ranged from 25 times more patients than features to 240 times more features than patients (median of 2.2 times more patients than features). Feature reduction or adjustment for multiple testing was used in 51% of studies (29/57) and while 14% (8/57) relied on prospectively acquired data, none included plans for radiomics analysis in its prospective study protocol. Validation of radiomics signatures on independent validation datasets was performed in 5% (3/57) of the studies, only one of which employed an external dataset.

**Fig. 3:**
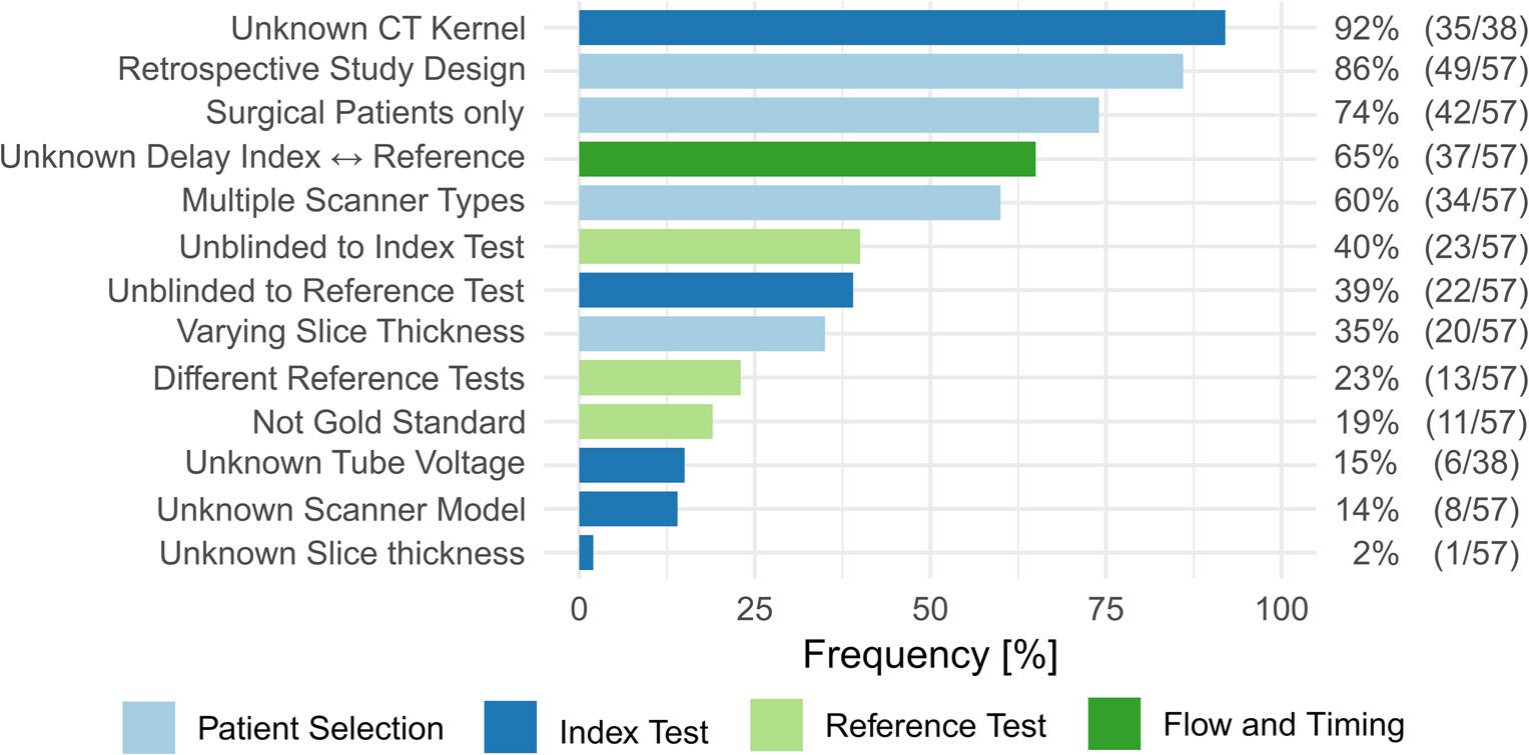
Risk factors for bias colored according the four dimensions of the QUADAS tool. The length of the bars is equivalent to the frequency with which this risk factor was identified among the included studies

Assessment of the studies with the QUADAS-2 tool revealed methodological aspects increasing the risk of bias. As QUADAS-2 is not intended as a quantitative score, concern of bias from the reviewers was aggregated qualitatively for the different dimensions addressed by the tool (Table S5). Risk factors for bias which were repeatedly identified are summarized in Fig. 3. Risk factors relating to patient selection and timing of index and reference tests were particularly frequently observed. Reporting the temporal delay between the index and reference test may be critical when determining tumor nuclear grade which influences progression and less critical when comparing RCC histological subtypes. The heavy reliance of literature on radiomics in RCC on retrospective surgical cohorts scanned with multiple scanners risks sampling technically variable data. Most studies explained texture feature extraction in detail; however, machine learning–based models were employed in many papers without sufficient description of the model parameters to allow replication.

The reproducibility of the RQS and QUADAS-2 was also assessed. During the training phase, particular variability in the rating of the detection and discussion of biological correlates was identified. The reviewers agreed to rate the item more liberally in agreement with previous publications [21]. The ICC for the RQS was 0.96 (95% CI 0.93–0.98). The ICC for studies rated by all three reviewers (11/57) was 0.92 (95% CI 0.80–0.98). Substantial or almost perfect agreement was achieved for most individual elements of the RQS. Only moderate agreement was reached in the assessment of the imaging protocol (Table 2). Absolute agreement concerning risk of bias and applicability of the seven indicator questions of the QUADAS tool was generally above 75% for most dimensions. Absolute agreement was 58% in the assessment of the risk of selection bias.

**Table 2:** Inter-rater agreement in the assessment of the RQS

RQS scoring item	S* [95% CI]
Image Protocol	0.45 [0.20–0.67]
Multiple Segmentations	0.93 [0.82–1.00]
Phantom Study	1.00 [1.00–1.00]
Multiple Time Points	0.93 [0.82–1.00]
Feature Reduction	0.93 [0.82–1.00]
Non Radiomics	0.67 [0.49–0.85]
Biological Correlates	0.93 [0.82–1.00]
Cut-off	0.93 [0.82–1.00]
Discrimination and Resampling	0.82 [0.71–0.92]
Calibration	0.96 [0.89–1.00]
Prospective	1.00 [1.00–1.00]
Validation	1.00 [1.00–1.00]
Gold Standard	0.76 [0.63–0.88]
Clinical Utility	0.60 [0.38–0.82]
Cost-effectiveness	1.00 [1.00–1.00]
Open Science	1.00 [1.00–1.00]

*CI*: confidence interval, *RQS*: Radiomics Quality Score

Publication bias is a concern in radiomics studies in particular. Indeed, only 4/57 (7%) publications included in this review report non-significant outcomes, all analyzing the differentiation of AML and RCC. In the absence of prospective investigations with pre-defined study protocols, selective reporting of positive outcomes is a risk.

**Fig. 4:**
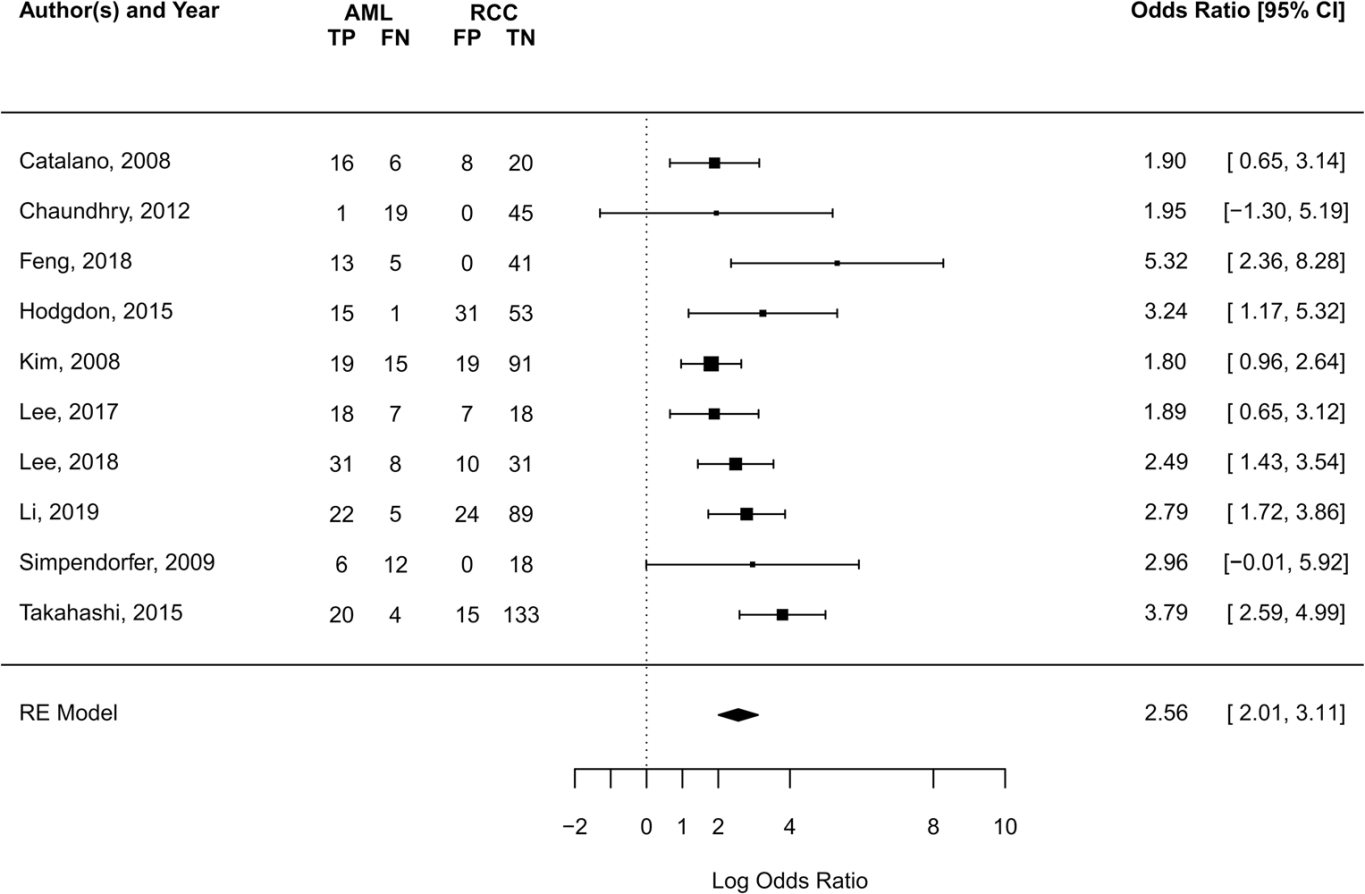
Forrest plot of the effect size calculated as log odds ratio for 10 of 13 studies investigating the diagnostic accuracy of radiomics in the differentiation of AMLwvf from RCC. TP: number of AMLwvf patients correctly diagnosed, FN: number of AMLwvf patients diagnosed as having RCC, FP: number of RCC patients diagnosed as having AML, TN: number of RCC patients correctly diagnosed. X-axis: log-transformed odds ratios, RE: random effects

**Fig. 5:**
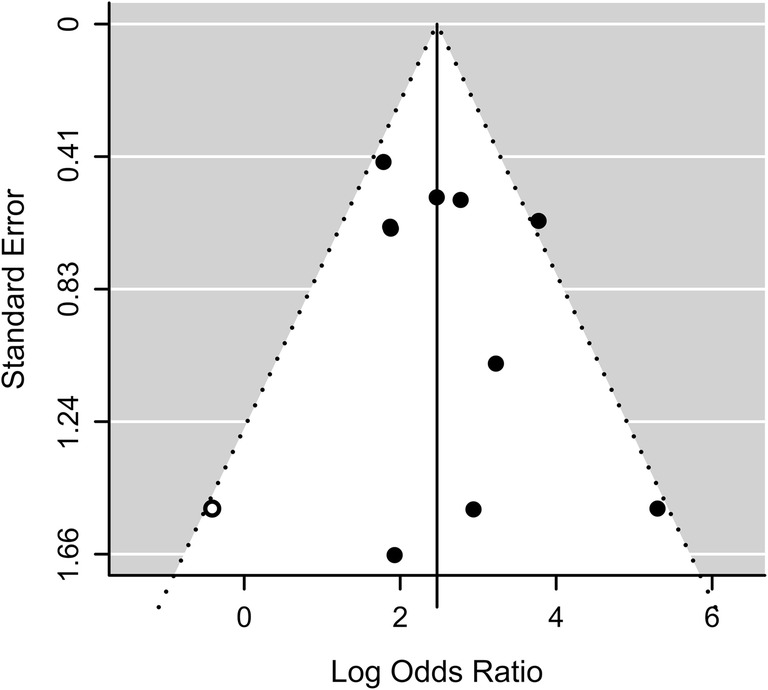
Funnel plot of studies included in the meta-analysis (black) and missing studies identified by trim and fill analysis (white dot). The funnel plot was asymmetric with more studies than expected reporting higher odds ratios for the ability of radiomics to differentiate between malignant renal tumors and benign AMLwvf; this can indicate the presence of publication bias

Thirteen of the 57 studies (23%) discussed the use of radiomics for the differentiation of AMLwvf and malignant renal tumors. Of these, 77% (10/13) provided information to reconstruct a contingency table and calculate the effect size and were included in the meta-analysis. The summary effect size under the random effects model across the studies indicated a diagnostic odds ratio of 5.89 (95% CI 4.02–8.23 *p* < 0.001) for radiomics models differentiating AMLwvf from RCC (Fig. 4). Cochran’s *Q* of 13.41, *p* = 0.15 with 9 degrees of freedom and *I*^2^ = 33.5% suggested the presence of moderate study-to-study dispersion. The funnel plot relating effect size to its standard error is shown in Fig. 5. Trim and fill analysis estimated that one study on the left side was missing. Following the addition of this study, the estimated overall effect size is OR = 5.55 (95% CI 3.77–8.16, *p* < 0.001). Considerable diversity existed among the radiomics features calculated and only mean in the unenhanced, entropy in the unenhanced and nephrographic phase CT were found to differentiate AMLwvf and RCC in two studies. Two studies assessing the ability of low attenuation voxel percentage to differentiate AMLwvf and RCC found significant differences in opposing directions [22, 23].

## Discussion

Radiomics may provide new quantitative imaging markers without the need to invest in new acquisition equipment or tracers. Multiple studies have shown promise in answering clinical questions that conventional, qualitative radiological diagnosis cannot answer. However, none of the multifactorial radiomics algorithms has achieved clinical translation or been independently validated. This systematic review has identified several common characteristics among the included studies that hinder rapid adoption of proposed algorithms into the clinic. Replication and independent validation of research findings relies on sharing of imaging data, segmentations, and code. None of the studies included in this review have provided open access to the code employed for data preparation, feature extraction, and model construction. This is particularly crucial where image pre-processing and artificial intelligence–based modeling were applied. Guidelines recommending reporting standards for machine learning (ML) models have been published; however, making the code used for data analysis publically available would be preferable [24]. Overall, 34/57 studies used ML models. There was a trend for these studies to be more recent than those not using any ML models. Furthermore, studies incorporating ML algorithms achieved significantly higher RQS ratings than studies without (5.16 ± 3.66 vs. 0.83 ± 4.27, *p* < 0.001). This was due, in particular, to less frequent validation of results, inclusion of non-radiomics parameters, and use of feature reduction and correction for multiple comparisons in non-ML studies.

Where patient numbers are limited and countless radiomics features can be quantified, it is critical to reduce the feature space, e.g., through removal of poorly reproducible features to reduce the risk of overfitting. This could be achieved with texture phantoms that were not employed by any of the studies in this review. Furthermore, appropriate statistical correction for multiple comparisons and independent validation, which has only been applied very rarely among the included studies, will reduce the risk of false positive and overly optimistic results. Meanwhile, prospective trials, where hypotheses are defined in advance, reduce the risk of reporting bias. Most trials included in this review only assessed surgical patients. However, surgical cohorts may be enriched in malignant lesions and larger tumor sizes, leading to selection bias. Small renal masses, which can be difficult to classify, may be assigned to active surveillance and are, therefore, underrepresented in surgical cohorts.

The RQS has been proposed to assess the methodological quality of radiomics studies, which is important to critically appraise the large number of publications and to prioritize validation of high-quality results. Because varying inter-rater agreement was observed in the first application of the RQS [21], two articles were used to train researchers. As a result, high agreement for the overall rating (ICC = 0.96) and most elements of the score (S* > 0.75) was achieved. Compared to the first application of the RQS, the average RQS rating was lower (10.8% vs 21.9%) as was the rating for the best performing study (48% vs 55.5%). Another, recently published review employing the RQS did not report inter-rater agreement [25].Only few systematic reviews in radiomics literature have been published and even fewer assessed methodological quality systematically and quantitatively. As a result, the RQS has not yet found widespread application.

The dependency of multiple radiomics features on image acquisition parameters has been demonstrated repeatedly [26–28]. However, only half of the studies included in this review documented the most important parameters. The selection of the scanner manufacturer and model, acquisition, and reconstruction parameters cause heterogeneity of imaging data. If the aim is to achieve broadly applicable radiomics models, standardization will be required wherever possible. Elsewhere, feature selection could consider robustness to variations in acquisition parameters and adjustments could be applied to the input data or the extracted features. The non-quantitative nature of T_1_- and T_2_-weighted MR sequences introduces additional heterogeneity even when acquisition parameters are kept constant. As a result, MR-based radiomics models frequently employed parametric maps, which do not require initial signal intensity normalization, were used most commonly. Out of 17 studies using MR, nine included the advanced diffusion coefficient based on diffusion-weighted imaging and two the transfer constant k^Trans^ from dynamic contrast-enhanced MRI in their analysis. Two studies employed ADC histogram parameters to differentiate tumor subtypes observing similar trends but differential statistical significance due to low numbers of cases.

Most studies segmented only part of the tumor. In light of recent findings highlighting the intratumoral heterogeneity in RCC on a genetic and metabolic level, texture analysis in a single 2D slice risks underestimating intratumoral heterogeneity [29–31]. However, studies segmenting single 2D slices of the tumor achieved equal RQS ratings and no trend over time favoring one segmentation strategy was apparent. The few publications comparing 2D and 3D texture analysis reached varying conclusions regarding their ability to correctly measure heterogeneity in tumors. However, it seems premature to suggest that segmentation of single slices was equivalent in diagnostic value to segmentation of an entire lesion. Only a small subset of the studies (5/57) placed small regions of interest within the tumor. These were either very early studies or studies where multiregional tissue sampling to match the regions of interest was carried out. Additionally, there is scope for further integration of radiomics data with clinical, genetic, and metabolic data to achieve a more complete understanding of renal cancer and harness the complementary value of each modality in cancer diagnostics, prognosis, treatment response prediction, and monitoring.

This review has some inherent limitations. First, the articles included in the meta-analysis differed slightly in their inclusion criteria. The control group was composed of ccRCC only in four studies while six included RCC of multiple subtypes. The methodology will always differ between radiomics studies as different centers use different equipment and the choice of image reconstruction, filtration, feature extraction, and calculation of radiomics models offer countless combinations. Still, a meta-analysis of the existing evidence provides important information as to the consistency of results and the magnitude of the effect size that can be anticipated and helps to estimate publication bias. Notably, the clinically more relevant question of differentiating oncocytoma from RCC was less frequently assessed. A number of studies included in this review were published before the introduction of the RQS. However, there was no trend for improvement over time; therefore, this was not thought to be a significant risk of bias. The RQS as well as QUADAS-2 have limitations. While the former is a quantitative metric and a debate about the appropriate weighting of different components is justified, the latter is a qualitative score and therefore less easily interpretable. Still, both scores are timely tools for the assessment of methodological quality of this highly specialized area of research.

In conclusion, radiomics models show promise for augmenting radiological diagnosis in renal cancer. The differentiation of AMLwvf and RCC has been investigated repeatedly and a meta-analysis showed moderate ability of radiomics to facilitate this distinction. However, well-designed and appropriately powered prospective radiomics trials are needed for these novel imaging markers to demonstrate their validity and progress towards clinical translation.

## Electronic supplementary material

ESM 1 (DOCX 102 kb).

## Abbreviations

AMLwvf: Angiomyolipoma without visible fat
ICC: Inter-rater correlation coefficient
ML: Machine learning
QUADAS: Quality Assessment of Diagnostic Accuracy Studies
RCC: Renal cell carcinoma
RQS: Radiomics Quality Score

## References

[1] Sullivan DC, Obuchowski NA, Kessler LG (2015). Metrology standards for quantitative imaging biomarkers. Radiology.

[2] Castellano G, Bonilha L, Li LM, Cendes F (2004). Texture analysis of medical images. Clin Radiol.

[3] Gillies RJ, Kinahan PE, Hricak H (2016). Radiomics: images are more than pictures, they are data. Radiology.

[4] Lubner Meghan G., Smith Andrew D., Sandrasegaran Kumar, Sahani Dushyant V., Pickhardt Perry J. (2017). CT Texture Analysis: Definitions, Applications, Biologic Correlates, and Challenges. RadioGraphics.

[5] Miles KA (2016). How to use CT texture analysis for prognostication of non-small cell lung cancer. Cancer Imaging.

[6] Ferlay J, Soerjomataram I, Dikshit R (2015). Cancer incidence and mortality worldwide: sources, methods and major patterns in GLOBOCAN 2012. Int J Cancer.

[7] Znaor Ariana, Lortet-Tieulent Joannie, Laversanne Mathieu, Jemal Ahmedin, Bray Freddie (2015). International Variations and Trends in Renal Cell Carcinoma Incidence and Mortality. European Urology.

[8] Pierorazio PM, Hyams ES, Mullins JK, Allaf ME (2012). Active surveillance for small renal masses. Rev Urol.

[9] Richard Patrick O., Lavallée Luke T., Pouliot Frederic, Komisarenko Maria, Martin Lisa, Lattouf Jean-Baptiste, Finelli Antonio (2018). Is Routine Renal Tumor Biopsy Associated with Lower Rates of Benign Histology following Nephrectomy for Small Renal Masses?. Journal of Urology.

[10] Defortescu G, Cornu J-N, Béjar S (2017). Diagnostic performance of contrast-enhanced ultrasonography and magnetic resonance imaging for the assessment of complex renal cysts: a prospective study. Int J Urol.

[11] Karlo CA, Di Paolo PL, Donati OF (2013). Renal cell carcinoma: role of MR imaging in the assessment of muscular venous branch invasion. Radiology.

[12] Hindman N, Ngo L, Genega EM (2012). Angiomyolipoma with minimal fat: can it be differentiated from clear cell renal cell carcinoma by using standard MR techniques?. Radiology.

[13] O’Connor JPB, Aboagye EO, Adams JE (2017). Imaging biomarker roadmap for cancer studies. Nat Rev Clin Oncol.

[14] McInnes MDF, Moher D, Thombs BD (2018). Preferred reporting items for a systematic review and meta-analysis of diagnostic test accuracy studies. JAMA.

[15] Lambin P, Leijenaar RTH, Deist TM (2017). Radiomics: the bridge between medical imaging and personalized medicine. Nat Rev Clin Oncol.

[16] Whiting PF, Rutjes AWS, Westwood ME (2011). QUADAS-2: a revised tool for the quality assessment of diagnostic accuracy studies. Ann Intern Med.

[17] R Core Team (2016) R: A Language and Environment for Statistical Computing. R Foundation for Statistical Computing. Available via http://www.r-project.org/. Accessed 31 Oct 2016

[18] Viechtbauer W (2010). Conducting meta-analyses in *R* with the metafor package. J Stat Softw.

[19] Marasini D, Quatto P, Ripamonti E (2016). Assessing the inter-rater agreement for ordinal data through weighted indexes. Stat Methods Med Res.

[20] Wang HY, Su ZH, Xu X et al (2016) Dynamic contrast-enhanced MR imaging in renal cell carcinoma: reproducibility of histogram analysis on pharmacokinetic parameters. Sci Rep 6. 10.1038/srep29146 10.1038/srep29146 PMC4933897 27380733

[21] Sanduleanu S, Woodruff HC, de Jong EECC (2018). Tracking tumor biology with radiomics: a systematic review utilizing a radiomics quality score. Radiother Oncol.

[22] Kim JY, Kim JK, Kim N, Cho K-S (2008). CT histogram analysis: differentiation of angiomyolipoma without visible fat from renal cell carcinoma at CT imaging. Radiology.

[23] Catalano OA, Samir AE, Sahani DV, Hahn PF (2008). Pixel distribution analysis: can it be used to distinguish clear cell carcinomas from angiomyolipomas with minimal fat?. Radiology.

[24] Jethanandani A, Lin TA, Volpe S (2018). Exploring applications of radiomics in magnetic resonance imaging of head and neck cancer: a systematic review. Front Oncol.

[25] Park Ji Eun, Kim Donghyun, Kim Ho Sung, Park Seo Young, Kim Jung Youn, Cho Se Jin, Shin Jae Ho, Kim Jeong Hoon (2019). Quality of science and reporting of radiomics in oncologic studies: room for improvement according to radiomics quality score and TRIPOD statement. European Radiology.

[26] Shafiq-ul-Hassan M, Zhang GG, Latifi K (2017). Intrinsic dependencies of CT radiomics features on voxel size and number of gray levels. Med Phys.

[27] Berenguer R, Pastor-Juan M d R, Canales-Vázquez J (2018). Radiomics of CT features may be nonreproducible and redundant: influence of CT acquisition parameters. Radiology.

[28] Zhao Binsheng, Tan Yongqiang, Tsai Wei Yann, Schwartz Lawrence H., Lu Lin (2014). Exploring Variability in CT Characterization of Tumors: A Preliminary Phantom Study. Translational Oncology.

[29] Gerlinger M, Rowan AJ, Horswell S (2012). Intratumor heterogeneity and branched evolution revealed by multiregion sequencing. N Engl J Med.

[30] Turajlic S, Xu H, Litchfield K (2018). Tracking cancer evolution reveals constrained routes to metastases: TRACERx renal. Cell.

[31] Okegawa T, Morimoto M, Nishizawa S (2017). Intratumor heterogeneity in primary kidney cancer revealed by metabolic profiling of multiple spatially separated samples within tumors. EBioMedicine.

