# Supplementary material for: Radiomics of computed tomography and magnetic resonance imaging in renal cell carcinoma—a systematic review and meta-analysis
Source: Eur Radiol. 2020 Feb 14;30(6):3558–66. doi: 10.1007/s00330-020-06666-3 (PMC7248043; doi:10.1007/s00330-020-06666-3)
Supplement: Supplementary file 1 — (DOCX 102 kb). [file 330_2020_6666_MOESM1_ESM.docx]

# Supplementary Materials

Supplementary Table S1 Data Collection Instrument Definitions

| Bibliographical Information | Field | Explanation |
| --- | --- | --- |
|  | Title |  |
|  | Authors |  |
|  | Year |  |
|  | Journal |  |
|  | Volume |  |
|  | Issue |  |
|  | Page |  |
|  | StudyID | Internal Study ID (to be determined) |
|  | Imaging Technique | MR, CT or PET/MR |
| MRI Technique | MRISequence | T1, T2, ADC (only the one used for texture analysis) |
|  | MRIManufacturer | Siemens, GE, Toshiba… |
|  | MRIModel | Avanto, Sonata… |
|  | MRITE | Echo Time |
|  | MRITR | Repetition Time |
|  | MRIETL | Echo Train Length |
|  | MRIBValues | b-values for calculation of ADC map |
|  | MRIContrastPhase | Name of Contrast phase (i.e. portal venous) |
|  | MRIContrastTiming | Seconds after injection |
|  | MRIFlipAngle | Flip Angle |
|  | MRI Thickness | Slie Thickness |
|  | MRIRespiration | Respiratory Triggered, breath hold |
| CT Technique | CTEnhancement | unenhanced, enhanced or both |
|  | CTManufacturer | Siemens, GE, Toshiba… |
|  | CTModel | SOMATOM Force, Aquilon ONE… |
|  | CTKernel | Vendor specific (B31s) or general (soft, bone) |
|  | CTKVP | Tube voltage |
|  | CTMAS | Tube current (auto, 250mAs) |
|  | CTContrastPhaseName | arterial, portal venous… |
|  | CTTiming | bolus tracking, 25s… |
|  | CTSliceThickness | Slice Thickness |
| Parameters for Radiomics Quality Score Parameters for Radiomics Quality Score | Protocol clear | Is the imaging protocol reproducible? |
|  | Registration | of multiple phases (yes, no) |
|  | MultipleTimepoints | Was repeat imaging carried out for texture comparison? |
|  | SegmentationTechnique | manual, semi-automatic, automatic |
|  | SegmentationDescription | 2D or 3D segmentation |
|  | SegmentationMultiple | Has segmentation been performed by multiple readers (yes/no) |
|  | Phantom | Has a phantom scan been performed (yes/no) |
|  | PhantomKind | What kind of phantom was used |
|  | PhantomParameters | Were the scanning parameters with the phantom identical to the patient parameters? |
|  | Multiple | Were multiple tests conducted? |
|  | MultipleMethodpValue | Were pValues adjusted for multiple testing? |
|  | MultipleMethodFeatureReduction | Was the number of features reduced to reduce the risk of false positives due to multiple testing? |
|  | MultipleNrCompFeatures | Number of features collected in total |
|  | MultipleNrSelFeatures | Number of features input in final model |
|  | Multivariate | Was a multivariate analysis performed? |
|  | MultivariateOutcomes | Is the multivariate analysis used for the predition of the primary outcome |
|  | MultivariatePredPower | Is the predictive power of the multivariate analysis reported? |
|  | MultivariateNonRad | Did the multivariate analysis include non-texture measurments (e.g. tumour volume) |
|  | BiologicalCorrelates | Was the model correlated to a biological endpoint (e.g. VHL mutation, VEGF expression) |
|  | CutOff | Was a cut off analysis performed? |
|  | CutOffprepost | Was the cut off selected prior to the analysis? |
|  | CutOffMethod | Where was the cut off selected (median, best discrimination)? |
|  | Discrimination | What discrimination statistic was used (AUC, Sensitivity/Specificity, none…) |
|  | Calibration | Was any kind fo calibration statistic used? |
|  | ProspectivePlan | Was the study planned prospectively? |
|  | ProspectiveData | Was the study data acquired prospectively? |
|  | RetrospectiveData | Was the data acquired retrospectively? |
|  | NrDatasets | Number of Patients in the study |
|  | NrCentres | How many centres does the data come from? |
|  | NrScanners | How many scanners were used to obtain the data? |
|  | Validation | Was a validation cohort used? |
|  | ValidationInternal | Was an internal (=same hospital) validation cohort used? |
|  | ValidationExternal | Was an external (=other hospital) validation cohort used? |
|  | ValidationExternalNrCohorts | How many external validation cohorts were used? |
|  | ValidationParamsConstant | Was the prediction model kept constant for the validation cohort? |
|  | GoldStandard | Was the gold standard used to define the dependent variable (e.g. Histology for tumur type)? |
|  | ClinicalApplications | Does the article mention clinical applications of texture analysis? |
|  | ClinicalApplicationsRelevance | Are these clinically relevant applications? |
|  | Cost-effectiveness | Was a cost analysis performed? |
| QUDAS | Representative | Is the participant sample representative of the population that will have the test afterwards? |
|  | Selection clear | Are the selection criteria clearly explained? |
|  | Reference Standard | Was a reference standard used to compare the texture model against? |
|  | Period to reference | Was the period between the texture analysis and the reference measurment short enough? |
|  | all verified | Have all participants been measured with a reference standard? |
|  | all same reference | Have all participants been measured with the same reference standard? |
|  | independent reference | Is the reference measurment independent of te texture measurments? |
|  | methods clear | Are the methods to obtain the texture parameters clear? |
|  | reference clear | Are the methods to obtain the reference standard clear? |
|  | blinded interpretation test | Was the person doing texture measurments blinded to the results of the reference test? |
|  | blinded interpretation reference | Was the person doing the reference test blinded to the results of texture measurmentst? |
|  | real world clinical data | Was real world clinical data available to the radiologist? |
|  | OpenScience | Were images, segmentations, code made available publicly? |
|  | PrimaryEndpoint | What was the primary endpoint of the study? |

Supplementary Table S2: Studies included in the systematic review. Bold studies included in the meta-analysis. AML: angiomyolipoma, AMLwvf: angiomyolipoma without visible fat, ccRCC: clear cell renal cell carcinoma, chRCC chromophobe renal cell carcinoma, Onc: Oncocytoma, pRCC: papillary renal cell carcinoma.

| **StudyID** | **Journal** | **#Pat** | **Modality** | **Research question** | **RQS (%)** |
| --- | --- | --- | --- | --- | --- |
| Antunes2016 | Transl Oncol | 2 | PET/MR | Detection of treatment response to antiangiogenic therapy | 8.5 |
| Bektas2019 | Eur Radiol | 53 | CT | Differentiation of low and high nuclear ccRCC | 5.7 |
| Bharwani2014 | Br. J. Cancer | 20 | MRI | Treatment-associated texture changes under sunitinib and correlation with overall survival | 7.5 |
| Bier2018 | PLoS ONE | 106 | CT | Prediction of tumour adherence to perinephric fat | 6.5 |
| Boos2017 | AJR Am J Roentgenol | 19 | CT | Correlation of CT texture to best response according to RECIST under VEGFR TKI | -4.0 |
| **Catalano2008** | **Radiology** | **50** | **CT** | **Differentiation of AMLwvf and ccRCC** | **-1.5** |
| Chandarana2012 | Radiology | 73 | MRI | Differentiation of ccRCC and pRCC | -0.5 |
| **Chaudhry2012** | **AJR Am J Roentgenol** | **64** | **CT** | **Differentiation of AMLwvf, ccRCC and pRCC** | **-1.0** |
| Chen2017 | Abdom Radiol (NY) | 94 | CT | Differentiation of ccRCC and oncocytoma | 4.5 |
| Chen2015 | Springerplus | 61 | CT | Differentiation of ccRCC and pRCC | 4.0 |
| Ding2018 | Eur J Radiol | 206 | CT | Differentiation of low and high nuclear grade ccRCC | 13.5 |
| Doshi2016 | AJR Am J Roentgenol | 37 | MRI | Differentiation of Type 1 and Type 2 pRCC | 6.0 |
| **Feng2018** | **Eur Radiol** | **58** | **CT** | **Differentiation of small AMLwvf and multiple RCC subtypes** | **6.5** |
| Gaing2015 | Invest Radiol | 44 | MRI | Differentiation of ccRCC, pRCC, chRCC, cysticRCC, oncocytoma and AML | 12.0 |
| Ghosh2015 | J Med Imaging (Bellingham) | 78 | CT | Prediction of BAP1 mutation status in ccRCC | 5.5 |
| Goh2011 | Radiology | 39 | CT | Correlation of CT texture at baseline and under TKI with time to progression | -1.7 |
| Haider2017 | Cancer Imaging | 40 | CT | Correlation with overall and progression-free survival under sunitinib | -2.0 |
| Hoang2018 | Abdom Radiol (NY) | 41 | MRI | Differentiation of small ccRCC, pRCC and oncocytoma | 4.5 |
| **Hodgdon2015** | **Radiology** | **100** | **CT** | **Differentiation of AMLwvf from multiple RCC subtypes** | **5.0** |
| Huhdanpaa2015 | Abdominal Imaging | 65 | CT | Differentiation of low and high nuclear grade ccRCC | -2.0 |
| Khene2018 | World J Urol | 70 | CT | Prediction of tumour adherence to perinephric fat | 4.5 |
| Kierans2014 | AJR Am J Roentgenol | 61 | MRI | Differentiation of low and high stage ccRCC | 5.5 |
| **Kim2008** | **Radiology** | **144** | **CT** | **Differentiation of AMLwvf from multiple RCC subtypes** | **-2.0** |
| Kocak2018 | Eur J Radiol | 93 | CT | Differentiation of ccRCC, pRCC and chRCC | 14.3 |
| Kunapuli2018 | J Digit Imaging | 150 | CT | Differentiation of benign and malignant renal lesions | 5.5 |
| **Lee2017** | **Med Phys** | **50** | **CT** | **Differentiation of AMLwvf and ccRCC** | **2.7** |
| **Lee2018** | **Med Phys** | **80** | **CT** | **Differentiation of AMLwvf and ccRCC** | **3.0** |
| Leng2017 | Abdom Radiol (NY) | 139 | CT | Differentiation of AMLwvf from multiple RCC subtypes | 0.0 |
| Li2018 | AJR Am J Roentgenol | 92 | MRI | Differentiation of AMLwvf, ccRCC, pRCC, chRCC, oncocytomas | 0.0 |
| **Li2019** | **Acad Radiol** | **140** | **MRI** | **Differentiation of AMLwvf and ccRCC** | **0.0** |
| Liu2017 | Int J Clin Exp Med | 44 | CT | Differentiation of ccRCC, pRCC, chRCC and pleomorphic sarcoma | 4.0 |
| Linguraru2011 | Med Phys | 43 | CT | Differentiation of renal lesions associated with VHL, BHD, HPRC and HLRCC | 5.0 |
| Lubner2016 | AJR Am J Roentgenol | 157 | CT | Correlation with overall survival, time to recurrence, histologic subtype, nuclear grade, sarcomatoid transformation and presence of metastasis | 3.5 |
| Mains2018 | Br J Radiol | 69 | CT | Correlation with overall and progression-free survival under various treatments | 7.0 |
| Paschall2018 | Abdom Radiol (NY) | 55 | MRI | Differentiation of ccRCC, pRCC and oncocytoma | -3.0 |
| Raman2014 | Acad Radiol | 99 | CT | Differentiation of ccRCC, pRCC, oncocytoma and cysts | 7.0 |
| Ramesh2018 | J Clin Diagn Res | 188 | CT | Differentiation of normal renal tissue, benign and malignant renal masses | -3.0 |
| Reynolds2018 | PLoS ONE | 12 | MRI | Delta radiomics for early prediction of response to radiotherapy | 4.5 |
| Sasaguri2015 | AJR Am J Roentgenol | 166 | CT | Differentiation of ccRCC and oncocytoma | 5.5 |
| Schieda2018 | AJR Am J Roentgenol | 37 | CT | Differentiation of low and high nuclear grade chRCC | 6.0 |
| Schieda2015 | AJR Am J Roentgenol | 31 | CT | Differentiation of ccRCC and sarcomatoid RCC | 5.5 |
| Scrima2019 | Abdom Radiol (NY) | 249 | CT | Differentiation of ccRCC and non-ccRCC, correlation with DNA expression levels | 3.5 |
| **Simpfendorfer2009** | **AJR Am J Roentgenol** | **36** | **CT** | **Differentiation of AMLwvf from RCC** | **0.0** |
| **Takahashi2015** | **AJR Am J Roentgenol** | **153** | **CT** | **Differentiation of AMLwvf from ccRCC** | **5.0** |
| Takahashi2016 | Abdom Radiol (NY) | 112 | CT | Differentiation of AML from ccRCC | -2.5 |
| Tanaka2011 | Int J Urol | 41 | MRI | Differentiation of AMLwvf from RCC | -3.0 |
| Varghese2018AJR | AJR Am J Roentgenol | 174 | CT | Differentiation of benign and malignant fat-poor renal masses | 5.7 |
| Varghese2018BJR | Br J Radiol | 156 | CT | Differentiation of benign and malignant fat-poor renal masses | -1.7 |
| Vendrami2018 | AJR Am J Roentgenol | 41 | MRI | Differentiation of type 1 and type 2 pRCC | 5.0 |
| Wang2016 | Sci Rep | 21 | MRI | Determination of reproducibility of DCE-based histogram parameters | 5.0 |
| Xi2018 | Eur Radiol | 16 | MRI | Differentiation of low and high nuclear grade ccRCC | 6.5 |
| Yan2015 | Acad Radiol | 48 | CT | Differentiation of AMLwvf, ccRCC and pRCC | 4.0 |
| Yap2018 | Urology | 150 | CT | Differentiation of benign and malignant predominantly solid renal masses | -1.7 |
| Yin2017 | Sci Rep | 9 | PET/MRI | Differentiation of the molecular subtypes ccA and ccB of ccRCC | 12.0 |
| Yin2018 | Clin Radiol | 8 | PET/MRI | Differentiation of the molecular subtypes ccA and ccB of ccRCC | 3.5 |
| Yu2017 | Abdom Radiol (NY) | 119 | CT | Differentiation of ccRCC, pRCC, chRCC and oncocytoma | 0.0 |
| Zhang2015 | AJR Am J Roentgenol | 46 | MRI | Differentiation of low and high nuclear grade ccRCC | -1.0 |

Supplementary Table S3: PRISMA Literature List. AML: angiomyolipoma, AMLwvf: angiomyolipoma without visible fat, BHD: Birt-Hogg-Dubé, BOLD: blood oxygenation level depentent, ccRCC: clear cell renal cell carcinoma, ceCT/MRI: contrast-enhanced CT/MRI, chRCC chromophobe renal cell carcinoma, DCE: dynamic contrast enhanced, DWI: diffusion weighted imaging, HLRCC: hereditary leiomyomatosis and renal cell carcinoma, HPRC: hereditary papillary renal carcinoma, Oncol: Oncology, pRCC: papillary renal cell carcinoma, Onc: Oncocytoma, Urol: Urology, VHL: von Hippel-Lindau.

| Study ID | Ref | Participant Characteristics | Clinical Setting | Study Design | Target Condition Definition | Index Test | Reference Standard | Sample Size | Funding Sources |
| --- | --- | --- | --- | --- | --- | --- | --- | --- | --- |
| Antunes2016 | (1) | metastatic RCC under sunitinib | Oncol | prospective clinical trial | metastatic RCC | PET/MR | Treatment application | 2 | public and corporate |
| Bektas2019 | (2) | ccRCC undergoing surgery, preoperative ceCT | Urol | retrospective | ccRCC | ceCT | Histological Fuhrman Grading | 53 | none |
| Bharwani2014 | (3) | untreated, histopathologically confirmed, metastatic ccRCC; Intermediate- and poor-risk | Uro  Oncol | retrospective analysis of prospective clinical trial data | metastatic ccRCC | MRI | Overall survival | 20 (DWI: 17) | NIHR |
| Bier2018 | (4) | ceCT under 3 months prior to laparoscopic partial nephrectomy with histological confirmation of RCC | Urol | retrospective | RCC | ceCT | Dindo-Clavien classification | 106 | none |
| Boos2017 | (5) | ceCT prior to VEGFR TKI therapy and at least two follow-ups, at least one RECIST target lesion | Oncol | retrospective | metastatic RCC | ceCT | RECIST 1.1 | 19 | not reported |
| Catalano2008 | (6) | Histopathologically proved AML or ccRCC with unenhanced CT. No visible fat on CT. | Urol | retrospective case-control | AMLwvf or ccRCC | CT | Histopathology | 50 | not reported |
| Chandarana2012 | (7) | Pathologic diagnosis of ccRCC or pRCC, ceMRI up to 180 days before surgery | Urol | retrospective | ccRCC or pRCC | ceMR | Histopathology | 73 | not reported |
| Chaudhry2012 | (8) | Histopathologically confirmed AMLwvf, ccRCC or pRCC, unenhanced CE | Urol | retrospective case-control | AMLwvf, ccRCC or pRCC | CT | Histopathology | 64 | not reported |
| Chen2017 | (9) | multiphase ceCT, undergoing nephrectomy for Onc or ccRCC | Urol | retrospective | Onc or ccRCC | ceCT | Histopathology | 94 | Whittier foundation, NCI |
| Chen2015 | (10) | multiphase ceCT, undergoing robotic partial nephrectomy for localized ccRCC or pRCC | Urol | retrospective | ccRCC or pRCC | ceCT | Histopathology | 61 | Whittier foundation, NCI |
| Ding2018 | (11) | triple phase ceCT undergoing resection of ccRCC | Urol | retrospective | ccRCC | ceCT | Histopathology | 206 | Public |
| Doshi2016 | (12) | preoperative MRI and nephrectomy for pRCC | Urol | retrospective | pRCC Type 1/2 | MRI | Histopathology | 37 | not reported |
| Feng2018 | (13) | Pathologically confirmed renal mass ≤4cm following radical or partial nephrectomy with preoperative triple phase CT | Urol | retrospective | AMLwvf, ccRCC pRCC, chRCC, cystic RCC | ceCT | Histopathology | 58 | none |
| Gaing2015 | (14) | Patients undergoing preoperative MRI for evaluation of renal masses | Urol | prospective imaging trial | ccRCC, pRCC, chRCC, cystic RCC, Onc, AML | IVIM MRI | Histopathology | 44 | RSNA |
| Ghosh2015 | (15) | Patients with ccRCC from TCGA | N/A | retrospective | ccRCC ± BAP1 mutation | ceCT | cBioPortal | 78 | UT MD Anderson Cancer Center NIH/NCI |
| Goh2011 | (16) | Metastatic RCC undergoing TKI treatment as first- or second-line therapy, TKI naïve, ceCT up to 4 weeks before start of therapy and following treatment | Oncol | retrospective | ccRCC, pRCC, sarcomatiod RCC | ceCT | Time to progression | 39 | not reported |
| Haider2017 | (17) | Metastatic ccRCC receiving Sunitinib as first of second line therapy, TKI naïve, ceCT up to 6 weeks before therapy and measurable disease according to RECIST 1.1 | Oncol | retrospective | metastatic ccRCC | ceCT | progression-free survival | 40 | Ontario institute for Caner Research |
| Hoang2018 | (18) | T1a renal lesions undergoing four phase MRI | Uro | retrospective | T1a RCC | ceMRI | Histopathology, genetic history, radiological opinion | 41 | NIH/NCI |
| Hodgdon2015 | (19) | Surgically resected AMLwvf or RCC <7 cm with preoperative unenhanced CT | Uro | retrospective case-controll | AMLwvf, ccRCC, pRCC, chRCC | CT | Histopathology | 100 | not reported |
| Huhdanpaa2015 | (20) | ceCT and pathology proven ccRCC | Uro | retrospective | ccRCC | CT | Histopathology | 65 | Whittier foundation, NCI |
| Khene2018 | (21) | Patients underwent robot-assisted partial nephrectomy | Uro | retrospective | renal masses | ceCT | Surgeon assessment | 70 | French Young Urological Association |
| Kierans2014 | (22) | Patients undergoing partial or total nephrectomy for renal neoplasm with MRI including DWI up to 180 days before surgery | Uro | retrospective | ccRCC | DWI MRI | Histopathology | 61 | not reported |
| Kim2008 | (23) | Unenhanced CT, pathologically proven AMLwf, RCC < 3.5cm or suspected AMLwvf in absence of growth for > 24 months | Uro | retrospective | AMLwvf, ccRCC, pRCC, chRCC | CT | Histopathology | 144 | not reported |
| Kocak2018 | (24) | Pathologically proven RCC following ceCT and TCGA data of ccRCC, pRCC and chRCC | Uro | retrospective | ccRCC, pRCC and chRCC | ceCT | Histopathology | 93 | None |
| Kunapuli2018 | (25) | Preoperative ceCT showing solid, enhancing, lipid poor tumours in patients undergoing resection | Uro | retrospective | ccRCC, pRCC, AMLwvf, Onc | ceCT | Histopathology | 150 | not reported |
| Lee2017 | (26) | Small renal masses, either ccRCC or AMLwvf | Uro | retrospective case-controll | ccRCC, AMLwvf | ceCT | Histopathology or Follow-up | 50 | National Research Foundation Korea, Seoul Women’s University |
| Lee2018 | (27) | Patients with AMLwvf or ccRCC | Uro | retrospective, possibly case-control | ccRCC, AMLwvf | ceCT | Histopathology or Follow-up | 80 | National Research Foundation Korea, Seoul Women’s University |
| Leng2017 | (28) | Patients with resected renal masses <4 cm, histopathological diagnosis of RCC or AMLwvf and CT prior to surgery | Uro | retrospective | RCC, AMLwvf | ceCT | Histopathology | 139 | None |
| Li2018 | (29) | Patients with resected renal masses ≤4 cm, histopathological diagnosis and DWI MRI | Uro | retrospective | ccRCC, pRCC, chRCC, AMLwvf | DWI MRI | Histopathology | 92 | not reported |
| Li2019 | (30) | Patients with resected renal masses ≤4 cm, histopathological diagnosis and DWI MRI | Uro | retrospective | ccRCC, AMLwvf | DWI MRI | Histopathology | 140 | National Natural Science Foundation China |
| Linguraru2011 | (31) | Patients with renal tumours and VHL disease, BHD syndrome, HPRC, HLRCC and ceCT | Uro | retrospective | VHL, BHD, HPRC, HLRCC | ceCT | unknown method | 43 | not reported |
| Liu2017 | (32) | Patients with sarcoma or RCC diagnosed by histopathology and with pre-operative ceCT | Uro | retrospective case-controll | primary undifferentiated pleomorphic sarcoma, ccRCC, pRCC, chRCC | ceCT | Histopathology | 44 | not reported |
| Lubner2016 | (33) | Patients undergoing surgical resection of renal tumours with prior ceCT. | Uro | retrospective | ccRCC, pRCC, chRCC, unclassified RCC | ceCT | Histopathology, overall survival | 157 | not reported |
| Mains2018 | (34) | Patients with metastatic RCC participating either an RCT or cohort study investigating angiogenesis inhibitors in mRCC. | Oncol | prospective | metastatic RCC | DCE-CT | overall and progression-free survival | 69 | Memorial Foun- dation of Eva and Henry Fraenkel, Health Research Fund of Central Denmark Region |
| Paschall2018 | (35 | Patient with renal lesions with a diameter >1cm imaged on one scanner. | Uro | retrospective | ccRCC, pRCC Type 1, Onc | DWI MRI | Histopathology (implicit) | 55 | NCI/NIH |
| Raman2014 | (36) | Patients undergoing surgical resection of ccRCC, pRCC, chRCC or Onc ≥2 cm and preoperative multiphase ceCT. | Uro | retrospective case-controll | ccRCC, pRCC, chRCC, Onc | ceCT | Histopathology | 80 | not reported |
| Ramesh2018 | (37) | Patients with ceCT and pathological confirmation of benign or malignant at least partially solid renal lesions. Indeterminate lesions excluded. | Uro | prospective | Partially solid or solid, benign and malignant renal lesions | ceCT | Histopathology of surgical specimen or percutaneous biopsy | 188 | not reported |
| Reynolds2018 | (38) | Patients with primary RCC, not eligible for surgery undergoing SABR and ceMRI | Oncol | Prospective non-randomized clinical trial | Primary RCC | DWI / DCE-MRI | Tumour volume on CT | 12 | ﻿Contributing to Australia Scholarship and Science Science and Medicine grant |
| Sasaguri2015 | (39) | Patients with RCC or Onc <4 cm and multiphase ceCT. Half of all RCC included, oncocytic neoplasm on biopsy excluded. | Uro | Retrospective | ccRCC, pRCC, chRCC, unclassified RCC, Onc | ceCT | Histopathology from surgery or biopsy | 166 | not reported |
| Schieda2018 | (40) | Patients with histologically confirmed chRCC and preoperative unenhanced CT | Uro | Retrospective case-control | chRCC | CT | Histopathology | 37 | not reported |
| Schieda2015 | (41) | Patients undergoing tumour resection with prior unenhanced CT | Uro | Retrospective case-control | ccRCC with and without sarcomatoid transformation | CT | Histopathology | 31 | not reported |
| Scrima2019 | (42) | Patients with ≤4 cm RCC undergoing unenhanced and pv ceCT followed by surgery without rhabdoid, sarcomatoid features and metastases. | Uro | retrospective | ccRCC, pRCC, chRCC, unclassified RCC | ceCT | Histopathology, Tissue microarray | 249 (Tissue Microarray: 41) | ﻿University of Wisconsin School of Medicine and Public Health Shapiro program and Depart- ment of Radiology Research and Development |
| Simpfendorfer2009 | (43) | Patients undergoing resection of renal mass for suspicion of RCC with prior triple phase CT. | Uro | retrospective, case-controll | RCC and AMLwvf | ceCT | Histopathology | 36 | not reported |
| Takahashi2015 | (44) | Patients with resected renal masses <4 cm, pathological diagnosis of RCC or AMLwvf and CT prior to surgery | Uro | retrospective | RCC, AMLwvf | ceCT | Histopathology | 153 | none |
| Takahashi2016 | (45) | Patients with resected renal masses <4 cm, pathological diagnosis of RCC or AMLwvf and unenhanced and ceCT prior to surgery | Uro | retrospective | RCC, AMLwvf | CT | Histopathology | 112 | not reported |
| Tanaka2011 | (46) | Patients undergoing DWI MRI and subsequent resection of fat-free renal tumours. | Uro | retrospective | ccRCC, AMLwvf | DWI MRI | Histopathology | 41 | not reported |
| Varghese2018 | (47) | Patients undergoing partial nephrectomy with preoperative ceCT | Uro | retrospective | ccRCC, pRCC, chRCC, AMLwvf, Onc | ceCT | Histopathology | 174 | Whittier Foundation |
| Varghese2018a | (48) | Patients undergoing surgical tumour resection with prior ceCT. | Uro | retrospective | ccRCC, pRCC, chRCC, AMLwvf, Onc | ceCT | Histopathology | 156 | Whittier Foundation |
| Vendrami2018 | (49) | Patients undergoing partial or radical nephrectomy post-surgically diagnosed as pRCC and pre-operative MRI. | Uro | retrospective | pRCC Type 1 and 2 | MRI | Histopathology | 41 | not reported |
| Wang2016 | (50) | Patients with suspicion of RCC on imaging, ≥18 years, GFR >60 m/s, Tumour > 1 cm, ccRCC. | Uro | Prospective | ccRCC | DCE-MRI |  | 21 | National Natural Science Foundation of China |
| Xi2018 | (51) | Patients with pT1b cRCC undergoing presurgical MRI | Uro | Prospective cross-sectional study | ccRCC | DCE-MRI | Histopathology | 18 | NIH |
| Yan2015 | (52) | Patients with resected, histologically confirmed AMLwvf and pre-operative unenhanced and ceCT. Matched ccRCC and pRCC cohorts. | Uro | Retrospective, case-control | ccRCC, pRCC, AMLwvf | ceCT | Histopathology from surgery or biopsy | 48 | National Scientific Foundation of China |
| Yap2018 | (53) | Patients with resected, solid, nonmacroscopic fat containing renal masses on multiphase CT. | Uro | Retrospective, case-control | ccRCC, pRCC, chRCC, AMLwvf, Onc | ceCT | Histopathology | 150 | not reported |
| Yin2018 | (54) | Patients with radiographic confirmation of renal mass, scheduled for surgery. Exclusion of non-ccRCC patients following surgery | Uro | Prospective | ccRCC ccA and ccB molecular subtypes | FDG PET-MRI | RNA sequencing | 8 | NIH/NCI, National Institute of Environmental Health Sciences, Fundamental Research Funds for the Central University of China, Siemens |
| Yin2017 | (55) | Patients with radiographic confirmation of renal mass, scheduled for surgery. Exclusion of non-ccRCC patients following surgery | Uro | Prospective | ccRCC | FDG PET-MRI | RNA sequencing, Immuno-histochemistry | 9 | NIH/NCI, National Institute of Environmental Health Sciences, UNC University Cancer Research Fund, Siemens |
| Yu2017 | (56) | Patients undergoing tumour resection for renal lesions ≥2cm with portal-venous abdominal CT up to 6 months prior. | Uro | Retrospective | ccRCC, pRCC, AMLwvf, Onc | ceCT | Histopathology | 119 | none |
| Zhang2015 | (57) | Patients undergoing tumour resection with MRI including DWI and BOLD at 3T up to 3 months before surgery and ccRCC histology. | Uro | Retrospective | ccRCC | DWI and BOLD MRI | Histopathology | 46 | not reported |

Supplementary Table S4 Individual RQS Ratings (SU / AB / LB) and average rating per item

| StudyID | Image Protocol | Multiple Segmentations | Phantom Study | Multiple Timepoints | Feature Reduction | Non Radiomics | Biological Correlates | Cut Off | Discrimination/ Resampling | Calibration | Prospective | Validation | Gold Standard | Clinical Utility | Cost | Open Science |
| --- | --- | --- | --- | --- | --- | --- | --- | --- | --- | --- | --- | --- | --- | --- | --- | --- |
| Antunes2016 | 1/1/- | 0/0/- | 0/0/- | 1/1/- | -3/3/- | 1/0/- | 1/1/- | 0/0/- | 0/0/- | 0/0/- | 7/7/- | -5/-5/- | 0/2/- | 2/2/- | 0/0/- | 0/0/- |
| Bektas2018 | 0/1/0 | 1/1/1 | 0/0/0 | 0/0/0 | 3/3/3 | 0/0/0 | 1/1/1 | 0/0/0 | 2/2/2 | 0/0/0 | 0/0/0 | -5/-5/-5 | 2/0/2 | 2/2/2 | 0/0/0 | 0/0/0 |
| Bharwani2014 | 1/-/1 | 1/-/1 | 0/-/0 | 1/-/1 | -3/-/-3 | 0/-/0 | 1/-/1 | 1/-/0 | 0/-/0 | 0/-/0 | 7/-/7 | -5/-/-5 | 2/-/2 | 2/-/2 | 0/-/0 | 0/-/0 |
| Bier2018 | 0/1/- | 1/1/- | 0/0/- | 0/0/- | 3/3/- | 0/1/- | 1/1/- | 0/0/- | 2/1/- | 0/0/- | 0/0/- | -5/-5/- | 2/2/- | 2/2/- | 0/0/- | 0/0/- |
| Boos2017 | 0/-/0 | 0/-/0 | 0/-/0 | 0/-/0 | -3/-/-3 | 0/-/0 | 1/-/1 | 0/-/0 | 0/-/0 | 0/-/0 | 0/-/0 | -5/-/-5 | 0/-/2 | 2/-/2 | 0/-/0 | 0/-/0 |
| Catalano2008 | 0/-/1 | 0/-/0 | 0/-/0 | 0/-/0 | -3/-/-3 | 0/-/0 | 1/-/1 | 1/-/1 | 1/-/1 | 0/-/0 | 0/-/0 | -5/-/-5 | 0/-/2 | 2/-/2 | 0/-/0 | 0/-/0 |
| Chandarana2012 | 0/-/1 | 1/-/1 | 0/-/0 | 0/-/0 | -3/-/-3 | 0/-/0 | 1/-/1 | 0/-/0 | 1/-/1 | 0/-/0 | 0/-/0 | -5/-/-5 | 2/-/2 | 2/-/2 | 0/-/0 | 0/-/0 |
| Chaudhry2012 | 0/-/0 | 1/-/1 | 0/-/0 | 0/-/0 | -3/-/-3 | 0/-/0 | 1/-/1 | 1/-/1 | 1/-/1 | 0/-/0 | 0/-/0 | -5/-/-5 | 0/-/2 | 2/-/2 | 0/-/0 | 0/-/0 |
| Chen2015 | 0/-/0 | 0/-/0 | 0/-/0 | 0/-/0 | 3/-/3 | 0/-/0 | 1/-/1 | 0/-/0 | 1/-/1 | 0/-/0 | 0/-/0 | -5/-/-5 | 2/-/2 | 2/-/2 | 0/-/0 | 0/-/0 |
| Chen2017 | 0/-/1 | 0/-/0 | 0/-/0 | 0/-/0 | 3/-/3 | 0/-/0 | 1/-/1 | 0/-/0 | 1/-/1 | 0/-/0 | 0/-/0 | -5/-/-5 | 2/-/2 | 2/-/2 | 0/-/0 | 0/-/0 |
| Ding2018 | 1/1/- | 1/1/- | 0/0/- | 0/0/- | 3/3/- | 0/1/- | 1/1/- | 0/0/- | 1/1/- | 0/0/- | 0/0/- | 2/2/- | 2/2/- | 2/2/- | 0/0/- | 0/0/- |
| Doshi2016 | 1/1/- | 1/1/- | 0/0/- | 0/0/- | 3/3/- | 0/0/- | 1/1/- | 0/0/- | 1/1/- | 0/0/- | 0/0/- | -5/-5/- | 2/2/- | 2/2/- | 0/0/- | 0/0/- |
| Feng2018 | 1/1/- | 1/1/- | 0/0/- | 0/0/- | 3/3/- | 0/0/- | 1/1/- | 0/0/- | 2/1/- | 0/0/- | 0/0/- | -5/-5/- | 2/2/- | 2/2/- | 0/0/- | 0/0/- |
| Gaing2015 | 1/-/1 | 1/-/1 | 0/-/0 | 0/-/0 | 3/-/3 | 0/-/0 | 1/-/1 | 0/-/0 | 0/-/0 | 0/-/0 | 7/-/7 | -5/-/-5 | 2/-/2 | 2/-/2 | 0/-/0 | 0/-/0 |
| Ghosh2015 | 0/0/- | 0/0/- | 0/0/- | 0/1/- | 3/3/- | 0/1/- | 1/1/- | 0/0/- | 2/2/- | 0/0/- | 0/0/- | -5/-5/- | 2/2/- | 0/2/- | 0/0/- | 0/0/- |
| Goh2011 | 1/1/0 | 0/0/0 | 0/0/0 | 0/0/0 | -3/-3/-3 | 0/1/0 | 1/0/0 | 0/0/0 | 1/1/1 | 0/0/0 | 0/0/0 | -5/-5/-5 | 2/2/2 | 2/2/2 | 0/0/0 | 0/0/0 |
| Haider2017 | 1/1/- | 0/0/- | 0/0/- | 0/1/- | -3/-3/- | 0/1/- | 1/1/- | 1/1/- | 0/0/- | 0/0/- | 0/0/- | -5/-5/- | 2/0/- | 0/2/- | 0/0/- | 0/0/- |
| Hoang2018 | 1/1/- | 0/0/- | 0/0/- | 0/0/- | 3/3/- | 0/0/- | 1/1/- | 0/0/- | 1/2/- | 0/0/- | 0/0/- | -5/-5/- | 2/0/- | 2/2/- | 0/0/- | 0/0/- |
| Hodgdon2015 | 0/0/- | 1/1/- | 0/0/- | 0/0/- | 3/3/- | 0/0/- | 1/1/- | 0/0/- | 1/1/- | 0/0/- | 0/0/- | -5/-5/- | 2/2/- | 2/2/- | 0/0/- | 0/0/- |
| Huhdanpaa2015 | 1/-/1 | 0/-/0 | 0/-/0 | 0/-/0 | -3/-/-3 | 0/-/0 | 1/-/1 | 0/-/0 | 0/-/0 | 0/-/0 | 0/-/0 | -5/-/-5 | 2/-/2 | 2/-/2 | 0/-/0 | 0/-/0 |
| Khene2018 | 0/0/- | 0/0/- | 0/0/- | 0/0/- | 3/3/- | 0/1/- | 1/1/- | 0/0/- | 1/1/- | 0/0/- | 0/0/- | -5/-5/- | 2/2/- | 2/2/- | 0/0/- | 0/0/- |
| Kierans2014 | 1/1/- | 1/1/- | 0/0/- | 0/0/- | 3/3/- | 0/1/- | 1/1/- | 0/0/- | 1/1/- | 0/0/- | 0/0/- | -5/-5/- | 2/2/- | 0/2/- | 0/0/- | 0/0/- |
| Kim2008 | 1/-/1 | 0/-/0 | 0/-/0 | 0/-/0 | -3/-/-3 | 0/-/0 | 1/-/1 | 0/-/0 | 1/-/1 | 0/-/0 | 0/-/0 | -5/-/-5 | 0/-/2 | 2/-/2 | 0/-/0 | 0/-/0 |
| Kocak2018 | 0/1/0 | 1/1/1 | 0/0/0 | 0/0/0 | 3/3/3 | 0/0/0 | 1/1/1 | 0/0/0 | 2/2/2 | 2/2/2 | 0/0/0 | 3/3/3 | 2/0/2 | 2/2/2 | 0/0/0 | 1/1/1 |
| Kunapuli2018 | 0/1/- | 0/0/- | 0/0/- | 0/0/- | 3/3/- | 0/0/- | 1/1/- | 0/0/- | 2/2/- | 0/0/- | 0/0/- | -5/-5/- | 2/2/- | 2/2/- | 0/0/- | 0/0/- |
| Lee2017 | 0/1/0 | 0/0/0 | 0/0/0 | 0/0/0 | 3/-3/3 | 0/0/0 | 1/1/1 | 0/0/0 | 2/2/1 | 0/0/0 | 0/0/0 | -5/-5/-5 | 2/0/2 | 2/2/2 | 0/0/0 | 0/0/0 |
| Lee2018 | 0/0/- | 0/0/- | 0/0/- | 0/0/- | 3/3/- | 0/0/- | 1/1/- | 0/0/- | 2/2/- | 0/0/- | 0/0/- | -5/-5/- | 0/0/- | 2/2/- | 0/0/- | 0/0/- |
| Leng2017 | 0/0/- | 0/0/- | 0/0/- | 0/0/- | 3/-3/- | 0/0/- | 1/1/- | 0/0/- | 1/1/- | 0/0/- | 0/0/- | -5/-5/- | 2/2/- | 0/2/- | 0/0/- | 0/0/- |
| Li2018 | 1/1/1 | 1/1/1 | 0/0/0 | 0/0/0 | -3/-3/-3 | 0/0/0 | 1/1/1 | 0/0/0 | 1/1/1 | 0/0/0 | 0/0/0 | -5/-5/-5 | 2/2/2 | 2/2/2 | 0/0/0 | 0/0/0 |
| Li2019 | 1/1/1 | 1/1/1 | 0/0/0 | 0/0/0 | -3/-3/-3 | 0/0/0 | 1/1/1 | 0/0/0 | 1/1/1 | 0/0/0 | 0/0/0 | -5/-5/-5 | 2/2/2 | 2/2/2 | 0/0/0 | 0/0/0 |
| Liu2017 | 0/1/- | 0/0/- | 0/0/- | 0/0/- | 3/3/- | 0/0/- | 1/1/- | 0/0/- | 1/0/- | 0/0/- | 0/0/- | -5/-5/- | 2/2/- | 2/2/- | 0/0/- | 0/0/- |
| Linguraru2011 | 0/-/0 | 0/-/0 | 0/-/0 | 0/-/0 | 3/-/3 | 0/-/0 | 1/-/1 | 0/-/0 | 2/-/2 | 0/-/0 | 0/-/0 | -5/-/-5 | 2/-/2 | 2/-/2 | 0/-/0 | 0/-/0 |
| Lubner2016 | 0/0/- | 0/0/- | 0/0/- | 0/0/- | 3/3/- | 0/0/- | 1/1/- | 0/0/- | 0/1/- | 0/0/- | 0/0/- | -5/-5/- | 2/2/- | 2/2/- | 0/0/- | 0/0/- |
| Mains2018 | 0/-/1 | 1/-/1 | 0/-/0 | 1/-/1 | -3/-/-3 | 0/-/0 | 1/-/1 | 0/-/0 | 1/-/0 | 0/-/0 | 7/-/7 | -5/-/-5 | 2/-/2 | 2/-/2 | 0/-/0 | 0/-/0 |
| Paschall2018 | 0/-/0 | 0/-/0 | 0/-/0 | 0/-/0 | -3/-/-3 | 0/-/0 | 1/-/1 | 0/-/0 | 1/-/1 | 0/-/0 | 0/-/0 | -5/-/-5 | 0/-/2 | 2/-/2 | 0/-/0 | 0/-/0 |
| Raman2014 | 1/1/- | 1/0/- | 0/0/- | 0/0/- | -3/-3/- | 0/0/- | 1/1/- | 0/0/- | 2/1/- | 0/0/- | 0/0/- | 2/2/- | 2/2/- | 2/2/- | 0/0/- | 0/0/- |
| Ramesh2017 | 0/0/- | 0/0/- | 0/0/- | 0/0/- | -3/-3/- | 0/0/- | 1/1/- | 0/0/- | 0/0/- | 0/0/- | 0/0/- | -5/-5/- | 2/2/- | 2/2/- | 0/0/- | 0/0/- |
| Reynolds2018 | 1/-/0 | 0/-/0 | 0/-/0 | 1/-/1 | -3/-/-3 | 0/-/0 | 1/-/1 | 0/-/0 | 0/-/0 | 0/-/0 | 7/-/7 | -5/-/-5 | 0/-/2 | 2/-/2 | 0/-/0 | 0/-/0 |
| Sasaguri2015 | 0/1/- | 0/0/- | 0/0/- | 0/0/- | 3/3/- | 1/1/- | 1/1/- | 0/0/- | 2/2/- | 0/0/- | 0/0/- | -5/-5/- | 2/2/- | 0/2/- | 0/0/- | 0/0/- |
| Schieda2015 | 0/1/- | 0/0/- | 0/0/- | 0/0/- | 3/3/- | 0/0/- | 1/1/- | 1/1/- | 1/1/- | 0/0/- | 0/0/- | -5/-5/- | 2/2/- | 2/2/- | 0/0/- | 0/0/- |
| Schieda2018 | 0/1/- | 1/1/- | 0/0/- | 0/0/- | 3/3/- | 0/1/- | 1/1/- | 0/0/- | 1/1/- | 0/0/- | 0/0/- | -5/-5/- | 2/2/- | 2/2/- | 0/0/- | 0/0/- |
| Scrima2018 | 0/0/- | 0/0/- | 0/0/- | 0/0/- | 3/3/- | 0/1/- | 1/1/- | 0/0/- | 0/0/- | 0/0/- | 0/0/- | -5/-5/- | 2/2/- | 2/2/- | 0/0/- | 0/0/- |
| Simpendorfer2009 | 0/-/0 | 1/-/1 | 0/-/0 | 0/-/0 | -3/-/-3 | 0/-/0 | 1/-/1 | 1/-/1 | 1/-/1 | 0/-/0 | 0/-/0 | -5/-/-5 | 2/-/2 | 2/-/2 | 0/-/0 | 0/-/0 |
| Takahashi2015 | 0/0/- | 0/0/- | 0/0/- | 0/0/- | 3/3/- | 1/1/- | 1/1/- | 0/0/- | 1/1/- | 0/0/- | 0/0/- | -5/-5/- | 2/2/- | 2/2/- | 0/0/- | 0/0/- |
| Takahashi2016 | 0/0/- | 0/0/- | 0/0/- | 0/0/- | -3/-3/- | 0/0/- | 1/1/- | 0/0/- | 1/0/- | 0/0/- | 0/0/- | -5/-5/- | 2/2/- | 2/2/- | 0/0/- | 0/0/- |
| Tanaka2011 | 0/-/0 | 0/-/0 | 0/-/0 | 0/-/0 | -3/-/-3 | 0/-/0 | 1/-/1 | 0/-/0 | 0/-/0 | 0/-/0 | 0/-/0 | -5/-/-5 | 2/-/2 | 2/-/2 | 0/-/0 | 0/-/0 |
| Varghese2018AJR | 0/1/1 | 1/1/1 | 0/0/0 | 0/0/0 | 3/3/3 | 0/0/0 | 1/1/1 | 0/0/0 | 1/1/1 | 0/0/0 | 0/0/0 | -5/-5/-5 | 2/2/2 | 2/2/2 | 0/0/0 | 0/0/0 |
| Varghese2018BJR | 0/1/0 | 0/0/0 | 0/0/0 | 0/0/0 | -3/-3/-3 | 0/0/0 | 1/1/1 | 0/0/0 | 1/1/1 | 0/0/0 | 0/0/0 | -5/-5/-5 | 2/2/2 | 2/2/2 | 0/0/0 | 0/0/0 |
| Vendrami2018 | 0/0/0 | 1/1/1 | 0/0/0 | 0/0/0 | 3/3/3 | 0/0/0 | 1/1/1 | 0/0/0 | 1/1/1 | 0/0/0 | 0/0/0 | -5/-5/-5 | 2/2/2 | 2/2/2 | 0/0/0 | 0/0/0 |
| Wang2016 | 1/-/0 | 1/-/1 | 0/-/0 | 0/-/0 | -3/-/-3 | 0/-/0 | 1/-/1 | 0/-/1 | 0/-/0 | 0/-/0 | 7/-/7 | -5/-/-5 | 0/-/2 | 2/-/2 | 0/-/0 | 0/-/0 |
| Xi2018 | 1/-/0 | 0/-/0 | 0/-/0 | 0/-/0 | -3/-/-3 | 0/-/0 | 1/-/1 | 0/-/0 | 2/-/2 | 0/-/0 | 7/-/7 | -5/-/-5 | 2/-/2 | 2/-/2 | 0/-/0 | 0/-/0 |
| Yan2015 | 1/1/1 | 0/0/1 | 0/0/0 | 0/0/0 | 3/3/3 | 0/0/0 | 1/1/0 | 0/0/0 | 1/0/1 | 0/0/0 | 0/0/0 | -5/-5/-5 | 2/0/2 | 2/2/2 | 0/0/0 | 0/0/0 |
| Yap2018 | 0/1/0 | 1/1/1 | 0/0/0 | 0/0/0 | -3/-3/-3 | 0/0/0 | 1/1/1 | 0/0/0 | 0/0/0 | 0/0/0 | 0/0/0 | -5/-5/-5 | 2/2/2 | 2/2/2 | 0/0/0 | 0/0/0 |
| Yin2017 | 1/1/- | 0/0/- | 0/0/- | 0/0/- | 3/3/- | 1/1/- | 1/1/- | 0/0/- | 0/0/- | 0/0/- | 7/7/- | -5/-5/- | 2/2/- | 2/2/- | 0/0/- | 0/0/- |
| Yin2018 | 0/0/- | 0/0/- | 0/0/- | 0/0/- | 3/3/- | 1/1/- | 1/1/- | 0/0/- | 1/0/- | 0/0/- | 0/0/- | -5/-5/- | 0/2/- | 2/2/- | 0/0/- | 0/0/- |
| Yu2017 | 1/1/- | 0/0/- | 0/0/- | 0/1/- | -3/-3/- | 0/0/- | 1/1/- | 0/0/- | 2/1/- | 0/0/- | 0/0/- | -5/-5/- | 2/2/- | 2/2/- | 0/0/- | 0/0/- |
| Zhang2015 | 1/-/1 | 0/-/0 | 0/-/0 | 0/-/0 | -3/-/-3 | 0/-/0 | 1/-/1 | 0/-/0 | 1/-/1 | 0/-/0 | 0/-/0 | -5/-/-5 | 2/-/2 | 2/-/2 | 0/-/0 | 0/-/0 |
| Average | 0.48 | 0.38 | 0.00 | 0.10 | 0.23 | 0.15 | 0.98 | 0.11 | 0.92 | 0.04 | 0.98 | -4.61 | 1.73 | 1.91 | 0.00 | 0.02 |
| Defined range | 0 – 2 | 0 – 1 | 0 – 1 | 0 – 1 | -3 – 3 | 0 – 1 | 0 – 1 | 0 – 1 | 0 – 2 | 0 – 1 | 0 – 7 | -5 – 5 | 0 – 2 | 0 – 2 | 0 – 1 | 0 – 4 |

Supplementary Table S5 QUADAS assessment for each study broken down by dimension and rater (SU / AB / LB)

| Study ID |  | Risk of Bias | | | | | | | | | | | | | | |  | Applicability Concern | | | | | | | | | | |
| --- | --- | --- | --- | --- | --- | --- | --- | --- | --- | --- | --- | --- | --- | --- | --- | --- | --- | --- | --- | --- | --- | --- | --- | --- | --- | --- | --- | --- |
|  |  | Patient Selection | | |  | Index Test | | |  | Reference Standard | | |  | Flow and Timing | | |  | Patient Selection | | |  | Index Test | | |  | Reference Standard | | |
| Antunes2016 |  | ☹ | ☹ | - |  | ? | ? | - |  | ☺ | ☺ | - |  | ☺ | ? | - |  | ☺ | ☺ | - |  | ☺ | ☺ | - |  | ☺ | ☺ | - |
| Bektas2019 |  | ☹ | ☺ | ☺ |  | ? | ☺ | ☺ |  | ☺ | ☺ | ☺ |  | ☺ | ☺ | ☺ |  | ☺ | ☺ | ☺ |  | ☺ | ☺ | ☺ |  | ☺ | ☺ | ☺ |
| Bharwani2014 |  | ☹ | - | ☺ |  | ☺ | - | ☺ |  | ☺ | - | ☺ |  | ☺ | - | ☺ |  | ☺ | - | ☺ |  | ☹ | - | ☺ |  | ☺ | - | ☺ |
| Bier2018 |  | ☺ | ☺ | - |  | ☺ | ☺ | - |  | ☺ | ☺ | - |  | ☺ | ☺ | - |  | ☺ | ☺ | - |  | ☺ | ☺ | - |  | ☺ | ☺ | - |
| Boos2017 |  | ☺ | - | ☺ |  | ☹ | - | ☹ |  | ☺ | - | ☺ |  | ? | - | ? |  | ☺ | - | ☺ |  | ☹ | - | ☹ |  | ☺ | - | ☺ |
| Catalano2008 |  | ☹ | - | ☹ |  | ? | - | ? |  | ☺ | - | ☺ |  | ? | - | ? |  | ☺ | - | ☺ |  | ☺ | - | ☺ |  | ☺ | - | ☺ |
| Chandarana2012 |  | ☹ | - | ☺ |  | ☹ | - | ☹ |  | ☺ | - | ☺ |  | ☺ | - | ☺ |  | ☺ | - | ☺ |  | ☺ | - | ☺ |  | ☺ | - | ☺ |
| Chaudhry2012 |  | ☹ | - | ☹ |  | ☺ | - | ☺ |  | ☺ | - | ☺ |  | ? | - | ? |  | ☺ | - | ☺ |  | ☺ | - | ☺ |  | ☺ | - | ☺ |
| Chen2017 |  | ☹ | - | ☺ |  | ☹ | - | ☹ |  | ☺ | - | ☺ |  | ? | - | ? |  | ☺ | - | ☺ |  | ☺ | - | ☺ |  | ☺ | - | ☺ |
| Chen2015 |  | ☹ | - | ☹ |  | ☹ | - | ☹ |  | ☺ | - | ☺ |  | ? | - | ? |  | ☺ | - | ☺ |  | ☺ | - | ☺ |  | ☺ | - | ☺ |
| Ding2018 |  | ☹ | ☺ | - |  | ☺ | ☺ | - |  | ☺ | ☺ | - |  | ? | ☺ | - |  | ☺ | ☺ | - |  | ☺ | ☺ | - |  | ☺ | ☺ | - |
| Doshi2016 |  | ☹ | ☺ | - |  | ☺ | ☺ | - |  | ☺ | ☺ | - |  | ? | ? | - |  | ☺ | ☺ | - |  | ☺ | ☺ | - |  | ☺ | ☺ | - |
| Feng2018 |  | ☹ | ☹ | - |  | ? | ☹ | - |  | ☺ | ☺ | - |  | ? | ☺ | - |  | ☺ | ☺ | - |  | ☺ | ☺ | - |  | ☺ | ☺ | - |
| Gaing2015 |  | ☹ | - | ☹ |  | ☺ | - | ☺ |  | ☺ | - | ☺ |  | ☺ | - | ☺ |  | ☺ | - | ☺ |  | ☺ | - | ☺ |  | ☺ | - | ☺ |
| Ghosh2015 |  | ☹ | ☺ | - |  | ☺ | ☺ | - |  | ☺ | ☺ | - |  | ? | ? | - |  | ☺ | ☺ | - |  | ☺ | ☺ | - |  | ☺ | ☺ | - |
| Goh2011 |  | ☺ | ☺ | ☺ |  | ☺ | ☺ | ☺ |  | ☺ | ☺ | ☺ |  | ☺ | ☺ | ☺ |  | ☺ | ☺ | ☺ |  | ☺ | ☺ | ☺ |  | ☺ | ☺ | ☺ |
| Haider2017 |  | ☺ | ☺ | - |  | ☺ | ☺ | - |  | ☺ | ☺ | - |  | ☺ | ☺ | - |  | ☺ | ☺ | - |  | ☺ | ☺ | - |  | ☺ | ☺ | - |
| Hoang2018 |  | ☹ | ☺ | - |  | ☺ | ☺ | - |  | ☹ | ☹ | - |  | ? | ? | - |  | ☺ | ☺ | - |  | ☺ | ☺ | - |  | ☺ | ☺ | - |
| Hodgdon2015 |  | ☹ | ☺ | - |  | ☺ | ☺ | - |  | ☺ | ☺ | - |  | ? | ? | - |  | ☺ | ☺ | - |  | ☺ | ☺ | - |  | ☺ | ☺ | - |
| Huhdanpaa2015 |  | ☹ | - | ☹ |  | ☺ | - | ☺ |  | ☺ | - | ☺ |  | ? | - | ? |  | ☺ | - | ☺ |  | ☺ | - | ☺ |  | ☺ | - | ☺ |
| Khene2018 |  | ☺ | ☺ | - |  | ? | ? | - |  | ☺ | ☺ | - |  | ☺ | ? | - |  | ☺ | ☺ | - |  | ☺ | ☺ | - |  | ☺ | ☺ | - |
| Kierans2014 |  | ☹ | ☺ | - |  | ☺ | ☺ | - |  | ☺ | ☺ | - |  | ☺ | ☺ | - |  | ☺ | ☺ | - |  | ☺ | ☺ | - |  | ☺ | ☺ | - |
| Kim2008 |  | ☹ | - | ☺ |  | ☹ | - | ☹ |  | ☹ | - | ☺ |  | ? | - | ? |  | ☺ | - | ☺ |  | ☺ | - | ☺ |  | ☺ | - | ? |
| Kocak2018 |  | ☺ | ☺ | ☺ |  | ☺ | ☺ | ☺ |  | ☺ | ☺ | ☺ |  | ☺ | ☺ | ☺ |  | ☺ | ☺ | ☺ |  | ☺ | ☺ | ☺ |  | ☺ | ☺ | ☺ |
| Kunapuli2018 |  | ☹ | ☹ | - |  | ☺ | ☹ | - |  | ☺ | ☺ | - |  | ? | ? | - |  | ☺ | ☺ | - |  | ☺ | ☺ | - |  | ☺ | ☺ | - |
| Lee2017 |  | ☺ | ☺ | ☺ |  | ? | ? | ? |  | ☺ | ☺ | - |  | ? | ? | ? |  | ☺ | ☺ | ☺ |  | ☺ | ☺ | ☺ |  | ☺ | ☺ | ☺ |
| Lee2018 |  | ☹ | ☺ | - |  | ? | ? | - |  | ☹ | ☹ | ☹ |  | ? | ? | - |  | ☺ | ☺ | - |  | ☺ | ☺ | - |  | ☺ | ☺ | - |
| Leng2017 |  | ☹ | ☺ | - |  | ☺ | ☺ | - |  | ☺ | ☺ | - |  | ? | ? | - |  | ☺ | ☺ | - |  | ☺ | ☺ | - |  | ☺ | ☺ | - |
| Li2018 |  | ☹ | ☹ | - |  | ☹ | ☹ | ☹ |  | ☺ | ☺ | ☺ |  | ? | ? | - |  | ☺ | ☺ | - |  | ☺ | ☺ | - |  | ☺ | ☺ | - |
| Li2019 |  | ☺ | ☺ | ☺ |  | ☹ | ☹ | ☹ |  | ☺ | ☺ | ☺ |  | ? | ? | ? |  | ☺ | ☺ | ☺ |  | ☺ | ☺ | ☺ |  | ☺ | ☺ | ☺ |
| Linguraru2011 |  | ☹ | - | ☹ |  | ? | - | ? |  | ☺ | - | ☺ |  | ? | - | ? |  | ☺ | - | ☺ |  | ☺ | - | ☺ |  | ☺ | - | ☺ |
| Liu2017 |  | ☹ | ☺ | - |  | ? | ? | - |  | ☺ | ☺ | - |  | ? | ? | - |  | ☺ | ☺ | - |  | ☺ | ☺ | - |  | ☺ | ☺ | - |
| Lubner2016 |  | ☹ | ☺ | - |  | ☹ | ☹ | - |  | ☺ | ☺ | - |  | ☺ | ☺ | - |  | ☺ | ☺ | - |  | ☺ | ☺ | - |  | ☺ | ☺ | - |
| Mains2018 |  | ☺ | - | ☺ |  | ? | - | ? |  | ☹ | - | ☺ |  | ? | - | ? |  | ☺ | - | ☺ |  | ☺ | - | ☺ |  | ☺ | - | ☺ |
| Paschall2018 |  | ☹ | - | ☹ |  | ☹ | - | ☹ |  | ☺ | - | ☺ |  | ☺ | - | ☺ |  | ☺ | - | ☺ |  | ☺ | - | ☺ |  | ☺ | - | ☺ |
| Raman2014 |  | ☹ | ☹ | - |  | ? | ? | - |  | ☺ | ☺ | - |  | ? | ? | - |  | ☺ | ☺ | - |  | ☺ | ☺ | - |  | ☺ | ☺ | - |
| Ramesh2018 |  | ☹ | ☹ | - |  | ? | ? | - |  | ☹ | ☺ | - |  | ? | ? | - |  | ☺ | ☺ | - |  | ☺ | ☺ | - |  | ☺ | ☺ | - |
| Reynolds2018 |  | ☹ | - | ☹ |  | ☹ | - | ? |  | ☹ | - | ☺ |  | ☺ | - | ☺ |  | ☺ | - | ☺ |  | ☺ | - | ☺ |  | ☺ | - | ☺ |
| Sasaguri2015 |  | ☹ | ☺ | - |  | ☹ | ☹ | - |  | ☺ | ☺ | - |  | ? | ? | - |  | ☺ | ☺ | - |  | ☺ | ☺ | - |  | ☺ | ☺ | - |
| Schieda2018 |  | ☹ | ☹ | - |  | ☺ | ☺ | - |  | ☺ | ☺ | - |  | ☹ | ☺ | - |  | ☺ | ☺ | - |  | ☺ | ☺ | - |  | ☺ | ☺ | - |
| Schieda2015 |  | ☹ | ☺ | - |  | ☺ | ☺ | - |  | ☺ | ☺ | - |  | ? | ? | - |  | ☺ | ☺ | - |  | ☺ | ☺ | - |  | ☺ | ☺ | - |
| Scrima2019 |  | ☹ | ☺ | - |  | ? | ? | - |  | ☺ | ☺ | - |  | ☺ | ☺ | - |  | ☺ | ☺ | - |  | ☺ | ☺ | - |  | ☺ | ☺ | - |
| Simpfendorfer2009 |  | ☹ | - | ☹ |  | ☺ | - | ☺ |  | ☺ | - | ☺ |  | ? | - | ? |  | ☺ | - | ☺ |  | ☺ | - | ☺ |  | ☺ | - | ☺ |
| Takahashi2015 |  | ☹ | ☺ | - |  | ☹ | ☹ | - |  | ☺ | ☺ | - |  | ? | ? | - |  | ☺ | ☺ | - |  | ☹ | ☹ | - |  | ☺ | ☺ | - |
| Takahashi2016 |  | ☹ | ☺ |  |  | ☹ | ☹ | - |  | ☺ | ☺ | - |  | ? | ? | - |  | ☺ | ☺ | - |  | ☺ | ☺ | - |  | ☺ | ☺ | - |
| Tanaka2011 |  | ☹ | - | ☹ |  | ☺ | - | ☺ |  | ☺ | - | ☺ |  | ? | - | ? |  | ☺ | - | ☺ |  | ☺ | - | ☺ |  | ☺ | - | ☺ |
| Varghese2018AJR |  | ☺ | ☺ | ☺ |  | ☺ | ☺ | ☺ |  | ☺ | ☺ | ☺ |  | ? | ? | ? |  | ☺ | ☺ | ☺ |  | ☺ | ☺ | ☺ |  | ☺ | ☺ | ☺ |
| Varghese2018BJR |  | ☺ | ☺ | ☺ |  | ? | ? | ? |  | ☺ | ☺ | ☺ |  | ? | ? | ? |  | ☺ | ☺ | ☺ |  | ☺ | ☺ | ☺ |  | ☺ | ☺ | ☺ |
| Vendrami2018 |  | ☹ | ☹ | ☹ |  | ☺ | ☺ | ☺ |  | ☺ | ☺ | ☺ |  | ? | ? | ? |  | ☺ | ☺ | ☺ |  | ☺ | ☺ | ☺ |  | ☺ | ☺ | ☺ |
| Wang2016 |  | ☺ | - | ☺ |  | ☹ | - | ☹ |  | ☺ | - | ☺ |  | ☺ | - | ☺ |  | ☺ | - | ☺ |  | ☺ | - | ☺ |  | ☺ | - | ☺ |
| Xi2018 |  | ☹ | - | ☺ |  | ☺ | - | ☺ |  | ☺ | - | ☺ |  | ☺ | - | ☺ |  | ☺ | - | ☺ |  | ☺ | - | ☹ |  | ☺ | - | ☺ |
| Yan2015 |  | ☹ | ☹ | ☹ |  | ☺ | ☺ | ☺ |  | ☹ | ☹ | ☺ |  | ? | ? | ? |  | ☺ | ☺ | ☺ |  | ☺ | ☺ | ☺ |  | ☺ | ☺ | ☺ |
| Yap2018 |  | ☹ | ☹ | ☹ |  | ☹ | ☹ | ? |  | ☺ | ☺ | ☺ |  | ? | ? | ? |  | ☺ | ☺ | ☺ |  | ☺ | ☺ | ☺ |  | ☺ | ☺ | ☺ |
| Yin2018 |  | ☹ | ☹ | - |  | ☹ | ☹ | - |  | ☺ | ☺ | - |  | ? | ? | - |  | ☺ | ☺ | - |  | ☺ | ☺ | - |  | ☺ | ☺ | - |
| Yin2017 |  | ☹ | ☺ | - |  | ☺ | ☺ | - |  | ☺ | ☺ | - |  | ☺ | ☺ | - |  | ☺ | ☺ | - |  | ☺ | ☺ | - |  | ☺ | ☺ | - |
| Yu2017 |  | ☹ | ☺ | - |  | ☺ | ☺ | - |  | ☺ | ☺ | - |  | ? | ? | - |  | ☺ | ☺ | - |  | ☺ | ☺ | - |  | ☺ | ☺ | - |
| Zhang2015 |  | ☹ | - | ☹ |  | ☹ | - | ☹ |  | ☺ | - | ☺ |  | ☺ | - | ☺ |  | ☺ | - | ☺ |  | ☺ | - | ☺ |  | ☺ | - | ☺ |

**Supplementary Statistical Methods**

Fleiss’ kappa statistic is used for the assessment of inter-rater agreement where a fixed number of raters has assigned a categorical value to a number of observations. As the Radiomics Quality Score includes categories with more than two possible ratings, a modified version of Fleiss’ kappa statistic was used which was adapted to measuring agreement for ordinal ratings. The ordinary kappa statistic depends on the prevalence of the outcome in the study population and on the number of possible ratings [58, 59]. The modified Fleiss’ kappa statistic proposed by Marasini *et al.* does not show this paradoxical behavior and has been implemented in the “raters” package for the R language for statistical computing. Additionally, the application of a Monte Carlo simulation allows estimating the confidence interval for the agreement through repeated random sampling (1000-times) on the one hand, as well as rejection of the null hypothesis that agreement may have arisen by chance alone. [60, 61].

The summed RQS score was treated as a continuous variable and inter-rater agreement assessed using the interclass correlation coefficient (ICC) with its implementation in the “irr” package for the R language for statistical computing [62]. Ten different forms of the ICC with different assumptions have been described. For the statistical analysis in this manuscript, the ICC using a single source, two-way random effects model has been employed. The two-way random effects model was chosen because a random subset of the same group of raters assessed each study and absolute agreement was measured as no systematic difference between raters should occur. Ratings from all raters were treated equally and, therefore, the mean was employed as the assessment basis [63].

The meta-analysis was conducted using the “metafor" package for R language for statistical computing [64]. Diagnostic odds ratios were calculated from contingency tables to derive the effect size. Due to the methodological heterogeneity between the studies included in this systematic review, a mixed effect model was chosen for the meta-analysis. In order to quantify the heterogeneity between the included studies, the Cochran’s Q and the I^2^ statistic were calculated. Cochran’s Q assesses the hypothesis that the distribution of results is homogenous and p-values < .05 would generally lead to the rejection of this null-hypothesis [65]. As with a small number of studies Cochran’s Q can be distorted, I^2^, an estimate of the percentage of the variability in effect estimators which can be attributed to true heterogeneity between studies rather than chance through sampling error, was also reported. I^2^ values of 25% and less are usually considered to be low or unimportant, 25% - 50% moderate and values above 75% are considered high [66]. Measuring inter-study dispersion assumes that, if all studies were methodologically identical and variation in results were only due to the random selection of study participants, the effect sizes would follow a chi-squared distribution. I^2^ is a measure for how much of the variability between effect size estimates is due to methodological heterogeneity rather than sampling error [67].

A funnel plot was employed as a tool to explore the presence of publication bias among the studies included in the meta-analysis. The logarithmic diagnostic odds ratio of an individual study is plotted against its standard error which is inversely related to sample size. An asymmetric distribution of studies on the funnel plot can be due to preferential reporting of significant studies and may therefore indicate publication bias. The dotted diagonal lines border the sector where 95% of all studies were assumed to lie [68]. Trim-and-fill analysis aims at identifying and correcting funnel plot asymmetry resulting from publication bias by removing small studies which contribute to the asymmetry and replacing them with their missing counterpart to achieve symmetry. This method performs better with decreasing inter-study dispersion [69].

**Supplementary References**

1. Antunes J, Viswanath S, Rusu M, et al (2016) Radiomics analysis on FLT-PET/MRI for characterization of early treatment response in renal cell carcinoma: A proof-of-concept study. Transl Oncol 9:155–162

2. Bektas CT, Kocak B, Yardimci AH, et al (2019) Clear Cell Renal Cell Carcinoma: Machine Learning-Based Quantitative Computed Tomography Texture Analysis for Prediction of Fuhrman Nuclear Grade. Eur Radiol 29:1153–1163

3. Bharwani N, Miquel ME, Powles T, et al (2014) Diffusion-weighted and multiphase contrast-enhanced MRI as surrogate markers of response to neoadjuvant sunitinib in metastatic renal cell carcinoma. Br J Cancer 110:616–624

4. Bier G, Bier S, Bongers MN, et al (2018) Value of computed tomography texture analysis for prediction of perioperative complications during laparoscopic partial nephrectomy in patients with renal cell carcinoma. PLoS One . doi: 10.1371/journal.pone.0195270

5. Boos J, Revah G, Brook OR, et al (2017) CT intensity distribution curve (Histogram) analysis of patients undergoing antiangiogenic therapy for metastatic renal cell carcinoma. Am J Roentgenol 209:W85–W92

6. Catalano OA, Samir AE, Sahani D V., Hahn PF (2008) Pixel Distribution Analysis: Can It be Used to Distinguish Clear Cell Carcinomas from Angiomyolipomas with Minimal Fat? Radiology 247:738–746

7. Chandarana H, Rosenkrantz AB, Mussi TC, et al (2012) Histogram Analysis of Whole-Lesion Enhancement in Differentiating Clear Cell from Papillary Subtype of Renal Cell Cancer. Radiology 265:790–798

8. Chaudhry HS, Davenport MS, Nieman CM, et al (2012) Histogram Analysis of Small Solid Renal Masses: Differentiating Minimal Fat Angiomyolipoma From Renal Cell Carcinoma. Am J Roentgenol 198:377–383

9. Chen F, Gulati M, Hwang D, et al (2017) Voxel-based whole-lesion enhancement parameters: a study of its clinical value in differentiating clear cell renal cell carcinoma from renal oncocytoma. Abdom Radiol 42:552–560

10. Chen F, Huhdanpaa H, Desai B, et al (2015) Whole lesion quantitative CT evaluation of renal cell carcinoma: differentiation of clear cell from papillary renal cell carcinoma. Springerplus 4:66

11. Ding J, Xing Z, Jiang Z, et al (2018) CT-based radiomic model predicts high grade of clear cell renal cell carcinoma. Eur J Radiol 103:51–56

12. Doshi AM, Ream JM, Kierans AS, et al (2016) Use of MRI in differentiation of papillary renal cell carcinoma subtypes: Qualitative and quantitative analysis. Am J Roentgenol 206:566–572

13. Feng Z, Rong P, Cao P, et al (2018) Machine learning-based quantitative texture analysis of CT images of small renal masses: Differentiation of angiomyolipoma without visible fat from renal cell carcinoma. Eur Radiol 28:1625–1633

14. Stoffel;Hersh BGSHBP, Sigmund EE, Huang WC, et al (2015) Subtype Differentiation of Renal Tumors Using Voxel-Based Histogram Analysis of Intravoxel Incoherent Motion Parameters. Invest Radiol 50:144–152

15. Ghosh P, Tamboli P, Vikram R, Rao A (2015) Imaging-genomic pipeline for identifying gene mutations using three-dimensional intra-tumor heterogeneity features. J Med Imaging 2:041009

16. Goh V, Nathan P, Juttla JK, et al (2011) Assessment of Response to Tyrosine Kinase Inhibitors in Metastatic Renal Cell Cancer: CT Texture as a Predictive Biomarker. Radiology 261:165–171

17. Haider MA, Vosough A, Khalvati F, et al (2017) CT texture analysis: A potential tool for prediction of survival in patients with metastatic clear cell carcinoma treated with sunitinib. Cancer Imaging 17:1–9

18. Hoang UN, Mojdeh Mirmomen S, Meirelles O, et al (2018) Assessment of multiphasic contrast-enhanced MR textures in differentiating small renal mass subtypes. Abdom Radiol 43:3400–3409

19. Hodgdon T, McInnes MDF, Schieda N, et al (2015) Can Quantitative CT Texture Analysis be Used to Differentiate Fat-poor Renal Angiomyolipoma from Renal Cell Carcinoma on unenhanced CT images. Radiology 276:787–796

20. Huhdanpaa H, Hwang D, Cen S, et al (2015) CT prediction of the Fuhrman grade of clear cell renal cell carcinoma (RCC): towards the development of computer-assisted diagnostic method. Abdom Imaging 40:3168–3174

21. Khene ZE, Bensalah K, Largent A, et al (2018) Role of quantitative computed tomography texture analysis in the prediction of adherent perinephric fat. World J Urol 36:1635–1642

22. Kierans AS, Rusinek H, Lee A, et al (2014) Textural Differences in Apparent Diffusion Coefficient Between Low- and High-Stage Clear Cell Renal Cell Carcinoma. Am J Roentgenol 203:W637–W644

23. Kim JY, Kim JK, Kim N, Cho K-S (2008) CT Histogram Analysis: Differentiation of Angiomyolipoma without Visible Fat from Renal Cell Carcinoma at CT Imaging. Radiology 246:472–479

24. Kocak B, Yardimci AH, Bektas CT, et al (2018) Textural differences between renal cell carcinoma subtypes: Machine learning-based quantitative computed tomography texture analysis with independent external validation. Eur J Radiol 107:149–157

25. Kunapuli G, Varghese BA, Ganapathy P, et al (2018) A Decision-Support Tool for Renal Mass Classification. J Digit Imaging . doi: 10.1007/s10278-018-0100-0

26. H.S. L, H. H, D.C. J, et al (2017) Differentiation of fat-poor angiomyolipoma from clear cell renal cell carcinoma in contrast-enhanced MDCT images using quantitative feature classification: Med Phys 44:3604–3614 . doi: 10.1002/mp.12258

27. Lee H, Hong H, Kim J, et al (2018) Deep feature classification of angiomyolipoma without visible fat and renal cell carcinoma in abdominal contrast-enhanced CT images with texture image patches and hand-crafted feature concatenation. Med Phys 45:1550–1561

28. Leng S, Takahashi N, Gomez Cardona D, et al (2017) Subjective and objective heterogeneity scores for differentiating small renal masses using contrast-enhanced CT. Abdom Radiol 42:1485–1492

29. Li A, Xing W, Li H, et al (2018) Subtype differentiation of small (≤ 4 cm) solid renal mass using volumetric histogram analysis of DWI at 3-T MRI. Am J Roentgenol 211:614–623

30. Li H, Li A, Zhu H, et al (2019) Whole-Tumor Quantitative Apparent Diffusion Coefficient Histogram and Texture Analysis to Differentiation of Minimal Fat Angiomyolipoma from Clear Cell Renal Cell Carcinoma. Acad Radiol 26:632–639

31. Linguraru MG, Wang S, Shah F, et al (2011) Automated noninvasive classification of renal cancer on multiphase CT. Med Phys 38:5738–5746

32. Liu G, Li W, Li L, Jiang X (2017) The value of CT image-based texture analysis for differentiating renal primary undifferentiated pleomorphic sarcoma from three subtypes of renal cell carcinoma. Int J Clin Exp Med 10:13526–13533

33. Lubner MG, Stabo N, Abel EJ, et al (2016) CT textural analysis of large primary renal cell carcinomas: Pretreatment tumor heterogeneity correlates with histologic findings and clinical outcomes. Am J Roentgenol 207:96–105

34. Mains JR, Donskov F, Petersen EM, et al (2017) Use of patient outcome endpoints to identify the best functional CT imaging parameters in metastatic renal cell carcinoma patients. Br J Radiol doi: 10.1259/bjr.20160795

35. Paschall AK, Mirmomen SM, Symons R, et al (2018) Differentiating papillary type I RCC from clear cell RCC and oncocytoma: application of whole-lesion volumetric ADC measurement. Abdom Radiol 43:2424–2430

36. Raman SP, Chen Y, Schroeder JL, et al (2014) CT texture analysis of renal masses: Pilot study using random forest classification for prediction of pathology. Acad Radiol 21:1587–1596

37. Ramesh G, Krishnamoorthy S, Sourirajan M, Sai V (2018) Assessment of primary solid renal mass using texture analysis of CT images of kidney by active contour method: A novel methody. J Clin Diagnostic Res

38. Reynolds HM, Parameswaran BK, Finnegan ME, et al (2018) Diffusion weighted and dynamic contrast enhanced MRI as an imaging biomarker for stereotactic ablative body radiotherapy (SABR) of primary renal cell carcinoma. PLoS One 13:e0202387

39. Sasaguri K, Takahashi N, Gomez-Cardona D, et al (2015) Small (< 4 cm) renal mass: Differentiation of oncocytoma from renal cell carcinoma on biphasic contrast-enhanced CT. Am J Roentgenol 205:999–1007

40. Schieda N, Lim RS, Krishna S, et al (2018) Diagnostic Accuracy of Unenhanced CT Analysis to Differentiate Low-Grade From High-Grade Chromophobe Renal Cell Carcinoma. Am J Roentgenol 210:1079–1087

41. Schieda N, Thornhill RE, Al-Subhi M, et al (2015) Diagnosis of sarcomatoid renal cell carcinoma with CT: evaluation by qualitative imaging features and texture analysis. Am J Roentgenol 204:1013–1023

42. Scrima AT, Lubner MG, Abel EJ, et al (2018) Texture analysis of small renal cell carcinomas at MDCT for predicting relevant histologic and protein biomarkers. Abdom Radiol 44:1999–2008

43. Simpfendorfer C, Herts BR, Motta-Ramirez GA, et al (2009) Angiomyolipoma with Minimal Fat on MDCT: Can Counts of Negative-Attenuation Pixels Aid Diagnosis? Am J Roentgenol 192:438–443

44. Takahashi N, Leng S, Kitajima K, et al (2015) Small (< 4 cm) renal masses: Differentiation of angiomyolipoma without visible fat from renal cell carcinoma using unenhanced and contrast-enhanced CT. Am J Roentgenol 205:1194–1202

45. Takahashi N, Takeuchi M, Sasaguri K, et al (2016) CT negative attenuation pixel distribution and texture analysis for detection of fat in small angiomyolipoma on unenhanced CT. Abdom Radiol 41:1142–1151

46. Tanaka HH, Yoshida S, Fujii Y, et al (2011) Diffusion-weighted magnetic resonance imaging in the differentiation of angiomyolipoma with minimal fat from clear cell renal cell carcinoma. Int J Urol 18:727–730

47. Varghese BA, Chen F, Hwang DH, et al (2018) Differentiation of predominantly solid enhancing lipid-poor renal cell masses by use of contrast-enhanced CT: Evaluating the role of texture in tumor subtyping. Am J Roentgenol 211:W288–W296

48. Varghese BA, Chen F, Hwang DH, et al (2018) Differentiating solid, non-macroscopic fat containing, enhancing renal masses using fast Fourier transform analysis of multiphase CT. Br J Radiol 20170789

49. Vendrami CL, Velichko YS, Miller FH, et al (2018) Differentiation of papillary renal cell carcinoma subtypes on MRI: Qualitative and texture analysis. Am J Roentgenol 211:1234–1245

50. Wang HY, Su ZH, Xu X, et al (2016) Dynamic Contrast-enhanced MR Imaging in Renal Cell Carcinoma: Reproducibility of Histogram Analysis on Pharmacokinetic Parameters. Sci Rep 6:29146

51. Xi Y, Yuan Q, Zhang Y, et al (2018) Statistical clustering of parametric maps from dynamic contrast enhanced MRI and an associated decision tree model for non-invasive tumour grading of T1b solid clear cell renal cell carcinoma. Eur Radiol 28:124–132

52. Yan L, Liu Z, Wang G, et al (2015) Angiomyolipoma with Minimal Fat: Differentiation From Clear Cell Renal Cell Carcinoma and Papillary Renal Cell Carcinoma by Texture Analysis on CT Images. Acad Radiol 22:1115–1121

53. Yap FY, Hwang DH, Cen SY, et al (2018) Quantitative Contour Analysis as an Image-based Discriminator Between Benign and Malignant Renal Tumors. Urology 114: . doi: https://doi.org/10.1016/j.urology.2017.12.018

54. Yin Q, Hung S-CC, Rathmell WKK, et al (2018) Integrative radiomics expression predicts molecular subtypes of primary clear cell renal cell carcinoma. Clin Radiol 73:782–791

55. Yin Q, Hung S-C, Wang L, et al (2017) Associations between Tumor Vascularity, Vascular Endothelial Growth Factor Expression and PET/MRI Radiomic Signatures in Primary Clear-Cell–Renal-Cell-Carcinoma: Proof-of-Concept Study. Sci Rep 7:43356

56. Yu HS, Scalera J, Khalid M, et al (2017) Texture analysis as a radiomic marker for differentiating renal tumors. Abdom Radiol 42:2470–2478

57. Zhang Y-D, Wu C-J, Wang Q, et al (2015) Comparison of Utility of Histogram Apparent Diffusion Coefficient and R2* for Differentiation of Low-Grade From High-Grade Clear Cell Renal Cell Carcinoma. Am J Roentgenol 205:W193–W201

58. Feinstein AR, Cicchetti D V. (1990) High agreement but low Kappa: I. the problems of two paradoxes. J Clin Epidemiol 43:543–549

59. Maclure M, Willett WC (1987) Misinterpretation and Misuse of the Kappa Statistic. Am J Epidemiol 126:161–169

60. Quatto P, Ripamonti E (2014) A Modification of Fleiss’ Kappa in Case of Nominal and Ordinal Variables. R package version 2.0.1

61. Marasini D, Quatto P, Ripamonti E (2016) Assessing the inter-rater agreement for ordinal data through weighted indexes. Stat Methods Med Res 25:2611–2633

62. Gamer M, Lemon J, Fellows I, Singh P (2019) irr: Various Coefficients of Interrater Reliability and Agreement. R package version 0.84.1

63. Koo TK, Li MY (2016) A Guideline of Selecting and Reporting Intraclass Correlation Coefficients for Reliability Research. J Chiropr Med 15:155–163

64. Viechtbauer W (2010) Conducting Meta-Analyses in R with the metafor Package. J Stat Softw. doi: 10.1103/PhysRevB.91.121108

65. Conover WJ (1999) Practical Nonparametric Statistics, 3rd ed. Wiley, New York

66. Higgins JPT, Thompson SG (2002) Quantifying heterogeneity in a meta-analysis. Stat Med 21:1539–1558

67. Higgins J, Thomas J, Chandler J, et al Cochrane Handbook for Systematic Reviews of Interventions version 6.0. https://training.cochrane.org/handbook/current/chapter-10. Accessed 17 Dec 2019

68. Sterne JAC, Egger M (2001) Funnel plots for detecting bias in meta-analysis: Guidelines on choice of axis. J Clin Epidemiol 54:1046–1055

69. Duval S, Tweedie R (2000) Trim and Fill: A Simple Funnel-Plot-Based Method of Testing and Adjusting for Publication Bias in Meta-Analysis. Biometrics 56:455–463
